# Postnatal developmental dynamics of cell type specification genes in Brn3a/Pou4f1 Retinal Ganglion Cells

**DOI:** 10.1186/s13064-018-0110-0

**Published:** 2018-06-29

**Authors:** Vladimir Vladimirovich Muzyka, Matthew Brooks, Tudor Constantin Badea

**Affiliations:** 1 Retinal Circuit Development & Genetics Unit, Building 6, Room 331B Center Drive, Bethesda, MD 20892–0610 USA; 2 Genomics Core, Neurobiology-Neurodegeneration & Repair Laboratory, National Eye Institute, NIH, Building 6, Room 331B Center Drive, Bethesda, MD 20892–0610 USA

**Keywords:** Retinal ganglion cell, Subtype specification, Postnatal development, Brn3a, RNA sequencing, in situ hybridization, Dendrite formation, Synapse formation

## Abstract

**Background:**

About 20–30 distinct Retinal Ganglion Cell (RGC) types transmit visual information from the retina to the brain. The developmental mechanisms by which RGCs are specified are still largely unknown. Brn3a is a member of the Brn3/Pou4f transcription factor family, which contains key regulators of RGC postmitotic specification. In particular, Brn3a ablation results in the loss of RGCs with small, thick and dense dendritic arbors (‘midget-like’ RGCs), and morphological changes in other RGC subpopulations. To identify downstream molecular mechanisms underlying Brn3a effects on RGC numbers and morphology, our group recently performed a RNA deep sequencing screen for Brn3a transcriptional targets in mouse RGCs and identified 180 candidate transcripts.

**Methods:**

We now focus on a subset of 28 candidate genes encoding potential cell type determinant proteins. We validate and further define their retinal expression profile at five postnatal developmental time points between birth and adult stage, using in situ hybridization (ISH), RT-PCR and fluorescent immunodetection (IIF).

**Results:**

We find that a majority of candidate genes are enriched in the ganglion cell layer during early stages of postnatal development, but dynamically change their expression profile. We also document transcript-specific expression differences for two example candidates, using RT-PCR and ISH. Brn3a dependency could be confirmed by ISH and IIF only for a fraction of our candidates.

**Conclusions:**

Amongst our candidate Brn3a target genes, a majority demonstrated ganglion cell layer specificity, however only around two thirds showed Brn3a dependency. Some were previously implicated in RGC type specification, while others have known physiological functions in RGCs. Only three genes were found to be consistently regulated by Brn3a throughout postnatal retina development – Mapk10, Tusc5 and Cdh4.

**Electronic supplementary material:**

The online version of this article (10.1186/s13064-018-0110-0) contains supplementary material, which is available to authorized users.

## Background

Retinal ganglion cells (RGCs) are the only neurons in the vertebrate retina which are directly connected to the brain. Mouse RGCs can be subdivided in ~ 30 different types with distinct specific molecular markers, dendritic arbor morphologies, synaptic partners and axonal projections [1–5]. There are several stages of RGC specification during embryonic and postnatal development. Mouse RGCs become postmitotic and start to express selective molecular markers at embryonic day 11 (E11). RGC axons pass the optic chiasm around E12 and reach the most remote retinorecipient areas of the brain by E15 [6, 7]. Around birth (postnatal day 0, P0) RGC axons invade their target nuclei in the brain, and the first two postnatal weeks are the most intense period for synapse formation. RGC dendrites begin their development later, and the lamination within the inner plexiform layer becomes clearly visible only around P3, grossly develops by P7, and reaches nearly final state after P14 [8, 9]. Mouse eyes open at P13-P14, when synapses between RGCs and bipolar and amacrine cells are already formed, and light-driven synaptic pruning takes place [10]. At P22 RGC axons and dendritic arbors are already “mature”, and cells are completely specified into multiple subtypes [11]. Numerous screens seeking to identify RGC subpopulation specific molecules were performed [12, 13], but we still do not have a comprehensive footprint of RGC diversity in terms of unique molecular signatures.

The Brn3/Pou4f family of transcription factors (TFs) comprising Brn3a, Brn3b and Brn3c is expressed in the retina specifically in RGCs, and its members form a major part of a combinatorial code for RGC type determination [1, 2, 11, 14, 15]. The RGC specification program requires Brn3b and/or Isl1 to initiate, and these TFs are crucial for survival, correct determination of cell fate and axonal targeting of RGCs during development [11, 16–23]. Brn3a starts to be expressed in embryonic retina 1 day later than Brn3b (E12.5 vs. E11.5). It is downstream of Brn3b in the RGC developmental transcriptional cascade, and was initially thought to function redundantly with Brn3b [24, 25]. Brn3a gene ablation is perinatal lethal due to the defects in the somatosensory system and brainstem nuclei, but it does not lead to dramatic perturbations within the retina at this point of development [26, 27]. A reporter knock-in allele expressing alkaline phosphatase (AP) at the Brn3a (*Brn3a*^*CKOAP*^) locus was previously used by our group to describe Brn3a cell type distribution among RGCs [2, 11], and also enabled us to identify axonal and dendrite arbor defects in RGCs missing Brn3a either alone or in combination with other Brn3s [28]. Dendrites of Brn3a-expressing RGCs are stratified in the outer laminae (~ 70%) of the inner plexiform layer (IPL) of the retina. Retina-specific Brn3a loss leads to an elimination of AP-expressing RGC arbors in a thin stripe along the border between ON and OFF layers of the IPL [11]. Retinal Brn3a loss results in a shift towards bistratified RGC dendritic arbor morphologies and overall RGC numbers reduction of around 30–40% depending on retinal region [11, 28], in part by eliminating small RGCs with dense multistratified dendritic morphology [2, 28]. The consequences of Brn3a ablation on RGC subpopulations are obvious already in the early postnatal period (P4) and associated with both cell fate change and cell number loss [11, 28]. However, the molecular mechanisms by which Brn3a controls RGC cell numbers and morphological features are still unclear. To find possible candidates of Brn3a-mediated regulation along with the novel subtype-specific markers, our group has recently performed a RNA deep sequencing screen which defined transcriptomes of Brn3a-positive RGCs and Brn3a-dependent RGC transcripts. The study found transcription factors (TFs), trans-membrane and intracellular structural molecules, that are enriched in RGC subpopulations and selectively depend on Brn3a expression. Overall, 180 transcripts were detected to be potential Brn3a targets at P3 [5].

Molecular determinants of neuronal subtypes can range from expression regulators to final-step effector molecules which provide a basis for cell-cell interactions and neuronal physiology and neuroanatomy [5]. TFs could serve as the most upstream “classifiers” of neuronal types by controlling the expression of identity-specific downstream genes which shape and modulate structural and functional characteristics of a cell [29–31]. Downstream effectors include cell-surface adhesion molecules which are crucial in definition of axon and dendritic morphologies via interaction with their partners on other cells [32–34]. Structural and cytoskeleton-associated molecules provide a mechanistic basis for morphological changes [35–38], and intracellular signaling molecules regulate those processes under transcription factor control [39–43]. Another key part of neuronal identity is provided by pre- and postsynaptic partners within the neuronal circuit. Specialized cell adhesion molecules, neurotransmitter and neurotrophin receptor subunits and other adaptors can confer specificity at both pre- and postsynaptic sites [40, 44–46]. Trophic factors and other secreted molecules are often secreted from postsynaptic sites to attract or repel presynaptic components (axons) and modulate connectivity during synaptogenesis and activity-dependent synaptic remodeling [47]. In addition to this, there are many non-secreted axon-guidance regulating molecules [48].

The goal of the current study is to identify potential Brn3a target molecules that contribute to the development and function of RGCs. Since we are interested in the terminal differentiation process – defining individual cell types – we are focusing on postnatal developmental time points. Specifically, we aim to analyze Brn3a influence on cell type determinants such as transcriptional regulators, adhesion molecules, synaptic elements and intracellular signaling/cytoskeleton apparatus. We used a Rax:Cre driver to produce retina-specific Brn3a ablation and evaluated potential targets previously identified in our P3 RNASeq screen by in situ hybridization (ISH), RT-PCR, and protein immunodetection experiments in postnatal Brn3a KO (Rax:Cre; *Brn3a*^*CKOAP/KO*^) and Brn3a WT (Rax:Cre; *Brn3a*^*CKOAP/WT*^) retinas. We performed our analysis over a developmental time series in order to correlate the expression dynamics of our target molecules to the postnatal stages of RGC development. We find that RNASeq and ISH data are in good agreement with regard to RGC enrichment and only to some extent to Brn3a regulation of our target genes at P3, but that many of the tested targets exhibit significant changes in expression profile between P0 and P22. For many of our target genes, multiple transcripts are expressed coincidentally in the retina with clearly distinct cell specificity. Finally, predicted intron-exon structures from genome annotation are not always accurate, and novel exon splicing sequences can be detected upon closer scrutiny. Nevertheless, multiple targets exhibit interesting patterns consistent with expression in one or a few RGC types, and regulation by Brn3a.

## Methods

### Mouse strains and crosses

Mouse lines carrying alleles Rax:Cre and *Brn3a*^*CKOAP*^ were previously described [11, 49]. To obtain Cre-mediated recombination in RGCs, the following cross was set up: Rax:Cre; *Brn3a*^*KO/WT*^ male x *Brn3a*^*CKOAP/CKOAP*^ female, to generate two types of progeny: Rax:Cre; *Brn3a*^*CKOAP/WT*^ and Rax:Cre; *Brn3a*^*CKOAP/KO*^. RGCs/retinas of these mice were either Brn3a heterozygotes, which are phenotypically wild type (*Brn3a*^*AP/WT*^, WT) or Brn3a knock-outs (*Brn3a*^*AP/KO*^, KO). All mice were on C57/Bl6-SV129 mixed background. All animal procedures were approved by the National Eye Institute (NEI) Animal Care and Use Committee under protocol NEI640.

### In situ hybridization (ISH)

ISH experiments were performed on P0, P3, P7, P14 and P22 eyes using an adapted Schaeren-Wiemers protocol [5]. Probes had a length between 160 and 900 bp (Table 1), a melting temperature between 77 °C and 94 °C, and were devoid of low-complexity regions, GC-rich or repetitive sequences. Probes for the main screen (experiments in Figs. 2, 3, 4, 5, 6, 7, 8, 9, 10, 11, 12 and 13) were targeted to 3’-UTRs of studied genes. Transcript-specific probes were designed for Pnkd and Clcc1 by targeting probes to unique exons as indicated in Figs. 14 and 15. The specific regions of the mRNA chosen for probes were in the areas highly covered by RNASeq reads, as determined from our data using IgViewer [50]. DNA templates for probes were derived by PCR from mouse genomic ES cell DNA (SV129 strain). All primers are provided in Table 1. A T3 promoter consensus sequence (GGAGCAAATTAACCCTCACTAAAGGG) was added to each reverse primer. Purified PCR products were used as templates for RNA probe synthesis, using T3 polymerase. Quality and concentration of the resulting RNA was assessed by 260/280 spectrometry or micro-electrophoretic analysis on a Bioanalyzer instrument (Thermo Scientific). Eyes for ISH were enucleated, cornea and lens removed, and the resulting eyecups were fixed for 1 h at RT in 4% paraformaldehyde (PFA). For each age, 4–8 eyes including Brn3a WT and KO genotypes were embedded in one OCT block, cryoprotected and sectioned at 14 μm thickness. ISH procedure was then performed as recently described [5], with two significant differences: 200 ng of probe were used for each hybridization, and the anti-DIG-AP antibody was used at 1:2000 (Fab fragments; Roche). The colorimetric reaction was developed between 2 and 9 h, individual incubation times for each probe are provided in Table 1. All images were acquired using the 20× objective of a Zeiss Axio Imager.Z2 at bright-field settings and Axiovision software. For each probe, genotype and age, six or more images derived from 2 to 3 animals were collected.

**Table 1:** Primers for ISH probes and RT-PCR

Gene	Forward Primer Sequence	Reverse Primer Sequence	Product Size, bp	Incubation (P0-P7), h	Incubation (P14-P22), h
For 3’-UTR probes					
Ankrd13b	TCCAAGGGCGGAGGCAGGT	ATCAGGGAAGGGAAGAGGAAG	821 + (26)	5	6
Cdh4	AAGTCCCAGCACTGATGAAAAA	CACCACCCGAATTGTTTGC	501 + (26)	6	6
Elfn1	CCTGCGCAAGAAGGTTCAGTT	CTTGCTTGCTTGCACCAGGC	367 + (26)	6	6
Eml1	CACTGTGATTTCTGTTTTGTCTA	GCCAGCCTCCCAAAGGGAG	473 + (26)	6	3
Fam19a4	AACTTTATGAACCTTGGAGAATG	TGAAATTGGAGGCAAGATGACT	498 + (26)	6	3
Foxp2	CTGTGCTGTTAGTGTAAAGATGT	GTTGCTTTCTAGAGTGTCATAAC	503 + (26)	3	3
Gabra1	GTTCTTTTAGTCGTATTCTGTTG	GAGCTTGCAAAATAGATTTGCC	900 + (26)	6	6
Grm4	GCCACACAGGCCTTCCTTCC	CTTCGAAACACACTCAAGATTAG	433 + (26)	6	5
Hpca	CCTCTCCCTCGTGTCTATCC	GAGCTGGGACAAGAAGTGTTC	443 + (26)	6	3
Plppr3	CAGTGCCAGCTCCGACTCTT	TTGCCAGGCTTAGTCCTGGTA	469 + (26)	6	6
Mapk10	GTCACAACGCACTCACGAAAG	ATCTGTATCTACATCCATCTGAC	364 + (26)	6	5
Nptx1	GGGGCTGAGAGCTCACTTG	ACCACTTCGAGCCACGCTC	600 + (26)	6	5
Nptx2	TCTCCGTCCCAGAGGCCAC	CAGTTCCCTCAGACGGAAAG	486 + (26)	6	6
Ntrk1	ATCGAGTGTATCACGCAGGGC	TATGATGGATGCTGGCCATGAA	328 + (26)	6	5
Pcdh20	GACGAGTTTCCTACTTCTTGGG	TTATCGTTGATGTCCAGAAGAAG	511 + (26)	6	9
Pick1	ACCACTGTAGGACAGCGAAGG	TTATTCAATACAGGCCCAGCTTC	393 + (26)	6	9
Pip5kl1	ACAAGGTGTGTCGAAGTCGAAG	TAAGGGTTGGGGTCAGGGTC	458 + (26)	6	6
Pnkd	CTTCACCATCCTCTTCATCACT	GAAGGTGGAAGATAGACTAGCC	360 + (26)	6	6
Rims1	CACCCTCCTCTGGAGTCCAG	TGTGTCTGCAGTTTATACCAATG	500 + (26)	2	3
Tmem25	TGCCTGTCCCTGTTGTGACC	GTACAAAACGATGCAGAGCTAC	687 + (26)	6	6
Tmem91	CAACAAGGCTTGGGCCAAGG	GGGTATCTTTAATTTCTCACATTG	348 + (26)	6	5
Tshz2	GCAGAAAGAAAGGGAAATATGTG	CATCACCTATTTGTTCTCTTCG	418 + (26)	6	6
Tusc5	CTGGAGATCTCCGAACCTACAT	ATGGAGGATTTCCGTATGGCC	492 + (26)	6	5
Pou4f1 (control)	TGTGTAGAAGATCCCCTTTGG	GCACAGAAATGGTTCTGATG	519 + (26)	3	3
For transcript-specific probes					
Clcc1 (pr.1 + pr.9) ^	CACAGCTGCGGGCCGAGC	CTGAGATTTCCTCATCGTTCCT	208 + (26)		
Clcc1 (pr.2 + pr.9)	AGTCCGCTCGGGACTCCAG	CTGAGATTTCCTCATCGTTCCT	181/252 + (26)	7	7
Clcc1 (pr.3 + pr.9) ^	TGTTTGAGGTAGGCGGCTCG	CTGAGATTTCCTCATCGTTCCT	220 + (26)		
Clcc1 (pr.4 + pr.9) ^	TCGGCGTCTTCCGCGGCC	CTGAGATTTCCTCATCGTTCCT	203 + (26)		
Clcc1 (pr.5 + pr.9) ^	TCCCTCTGAAAGAGCAGGCAG	CTGAGATTTCCTCATCGTTCCT	172 + (26)		
Clcc1 (pr.6 + pr.9) ^*	TGAGTGGGCGCTCTTCGGTG	CTGAGATTTCCTCATCGTTCCT	293 + (26)		
Clcc1 (pr.7 + pr.9) ^	CGTTTCCAGGATACACCGAGA	CTGAGATTTCCTCATCGTTCCT	300 + (26)		
Clcc1 (pr.2 + pr.8) ^*	AGTCCGCTCGGGACTCCAG	CGGGTTAAGGGAAGTCAAATTC	199/270 + (26)		
Clcc1(pr.7 + pr.8) ^^	CGTTTCCAGGATACACCGAGA	CGGGTTAAGGGAAGTCAAATTC	159 + (26)	4	3
Clcc1(pr.10 + pr.11)^	GTGAGGTCTGGAACATCAGAG	CATAAACACATTATATGGATCTA	432 + (26)		
Pnkd (pr.1 + pr.2)	TGGGACCCGAACATGGCGG	GGCTAGCCCCACGGCTTTC	246 (+ 26)	2	3
Pnkd (pr.3 + pr.4) *	TGTGGTATCCTCTTCTTCGTC	CTCCTTCCACGGTGTGGAAG	289 + (26)	2	3
Pnkd (pr.5 + pr.6)	CCCAGCATGGCTTGGCAGG	CCGATTCGGAAGAGCAGCCG	167 (+ 26)	2	3
Pnkd (pr.5 + pr.7) ^	CCCAGCATGGCTTGGCAGG	ATTGAAGAGGCGGGGCTGAG	282 (+ 26)		

Left column represents gene name, or gene name together with primer combination for transcript-specific probes. Second and third columns show primer sequences, fourth column shows product length, fifth and sixth columns represent ISH probe incubation times for P0-P7 and P14-P22 retinas. Some of the primer combinations could give rise to two different DNA fragments depending on splice variants. ^ - primer combination used for RT-PCR not ISH. ^^ - primer combination used for ISH not RT-PCR. * - primer combination gave negative result in RT-PCR. Primer combinations pr.3 + pr.8, pr.4 + pr.8, pr.6 + pr.8 for Clcc1 not shown in this table also gave negative results (for predicted fragment sizes see Table 2). Numbers in parenthesis refer to T3 promoter extension, as required for ISH probe generation

Quantitation: For each genotype and age 2–3 images were used. From each image for P7-P22 ages we picked 3 areas (ROIs) in each of GCL (ganglion cell layer), IPL (inner plexiform layer), INL (inner nuclear layer) and ONL (outer nuclear layer) and quantitated mean gray value of the pixels. The areas of ROIs from same retina layer were similar. For each measurement, GCL, INL, ONL and IPL ROIs of same length were registered along the section plane. At P3 we were restricted only to 3 layers (GCL, IPL and NBL - neuroblast layer), at P0 – only two – GCL and NBL. For P7-P22 measurements, we subtracted the GCL, the INL and the ONL values from the respective IPL ROI value to acquire normalized values. In case of P3 we subtracted the GCL and the NBL values from the IPL ones. Finally, in case of P0 we were not able to provide IPL normalization because at this age there are no visible IPL. Normalized (P3-P22) and un-normalized (P0) values we then used for representation on the plots.

There are certain limitations to our quantitation method especially in the case of sparsely expressed genes. Even if sparsely located cells have extremely high expression levels, after averaging to the whole area, this could be hidden by the non-expressing or low-expressing majority of cells (for example, in case of Gabra1 expression in WT GCL at P14).

### Reverse transcription – Polymerase chain reaction (RT-PCR)

Total RNA from P3 WT retinas was extracted as previously described. Reverse transcription was performed according to manufacturer’s instructions using SuperScript II RT (Invitrogen) and PCR amplification using Taq DNA polymerase (New England Biolabs), using a touch-down protocol. Forward and reverse primers for PCR are shown in Table 1, and primer combinations used for transcript-specific identification are presented in Table 2. PCR products were assessed in 1.5% agarose gel electrophoresis, extracted from gel, eluted in TE buffer, and inserted by T-A cloning into the pGEM-T Easy Vector System (Promega). After transformation, 4 colonies for each inserted PCR product were recovered, and analyzed by restriction digestion. All insert diagnostic digests resulted in DNA fragments of expected length. For each predicted product, identity was confirmed by sequencing plasmid DNA from two of the recovered colonies.

**Table 2:** Predicted and confirmed splicing variants for Clcc1 and Pnkd

Primer Pair	fragment size, bp	Predicted transcripts	Confirmed splice junctions
Clcc1			
pr.1 + pr.9	208 + (26)	NM_145543^	ex.1a_ex.3
pr.2 + pr.9	181+ (26), 252 + (26)	NM_145543^, NM_001177771, XM_006501416^, XM_011240107	ex.1a/1b_ex.3
pr.3 + pr.9	220 + (26)	NM_001177771, XM_006501416^, XM_011240107	ex.1b_ex.3
pr.4 + pr.9	203 + (26)	NM_001177771, NM_001177770, XM_006501416^, XM_011240107	ex.1b_ex.3
pr.5 + pr.9	172 + (26)	NM_001177771, NM_001177770, XM_006501416^, XM_011240107	ex.1b_ex.3
pr.6 + pr.9*	293 + (26)	NM_001177770, XM_011240107	none
pr.7 + pr.9	300 + (26)	NM_001177771, novel uncharacterized	ex.2_ex.3
pr.2 + pr.8*	199 + (26) 270 + (26)	NM_001177771	none
pr.3 + pr.8*	238 + (26)	NM_001177771	none
pr.4 + pr.8*	221 + (26)	NM_001177771	none
pr.6 + pr.8*	453 + (26)	none	none
pr.7 + pr.8	159 + (26)	NM_001177771, novel uncharacterized	ex.2
pr.10 + pr.11	432 + (26)	all transcripts	ex.4_ex.5_ex.6
Pnkd			
pr.1 + pr.2	246 + (26)	NM_025580, NM_001039509^	ex.2_ex.3
pr.3 + pr.4*	289 + (26)	NM_025580	none
pr.5 + pr.6	167 + (26)	NM_019999^	ex.5
pr.5 + pr.7	282 + (26)	NM_019999^	ex.5_ex.6
pr.8 + pr.9	360 + (26)	all transcripts	3’-UTR

Column 1 represents primer combinations, column 2 – DNA fragment size after RT-PCR. Some of the primer combinations could give rise to two different DNA fragments from two different splice variants. Column 3 shows transcript variants predicted to be recognized by the respective primer combination. Column 4 demonstrates exon-to-exon splicing junction which were experimentally confirmed by RT-PCR using respective primer combination. ^ - transcripts detected by RT-PCR. * - primer combination which gave negative result in RT-PCR. Numbers in parenthesis refer to T3 promoter extension, as required for ISH probe generation (Table should appear after “*Clcc1 transcripts detected in the retina”* section in “Results”)

### Indirect immunofluorescence (IIF)

For IIF, eyecups were fixed in 2% PFA for 30 min at RT, and cryoprotected in OCT. Slides were blocked (PBS, 10% bovine serum albumin (BSA), 10% normal donkey serum (NDS) and 0.5% Triton) for 1 h at RT and incubated at 4 °C overnight in primary antibody mixes (PBS, 10% BSA, 3% NDS, 0.5% Triton, and primary antibodies). Antibodies used were 1:100 rat anti-Cdh4 (MRCD5, provided by M. Takeichi and H. Matsunami to DSHB; dshb.biology.uiowa.edu; [51]), 1:200 rabbit anti-Elfn1 (gift from Dr. K. Martemyanov, The Scripps Research Institute, Jupiter, USA), 1:200 sheep anti-AP (American Research products, 13–2355), 1:20 mouse anti-Brn3a (MAB1585; Chemicon-Millipore). After washes, secondary antibody solutions were applied for 1 h RT (150 μl per slide - PBS, 10% BSA, 3% NDS, 0.5% Triton, 0.5 μl of 1000× DAPI and secondary antibodies). All secondaries were Alexa conjugated Donkey sera from Molecular Probes. Images were captured with a 40× lens on a Zeiss Imager.Z2 fitted with an Apotome for fluorescent imaging and Axiovision software. For each probe, genotype and age, eight or more images derived from at least 3 animals were collected.

### Statistical analysis

We used Kolmogorov-Smirnov (KS2) test for statistical assessment of ISH quantitation results. Sample numbers, means, medians and significance levels for KS2 and student t tests are provided in Additional file 1: Table S1.

## Results

### Screening strategy

To identify genes that could be responsible for RGC type specification in Brn3a-knockout retinas, we performed an in situ hybridization screen using a set of potential Brn3a-specific target genes recently identified in our RGC-specific RNASeq screening [5].

**Fig. 1:**
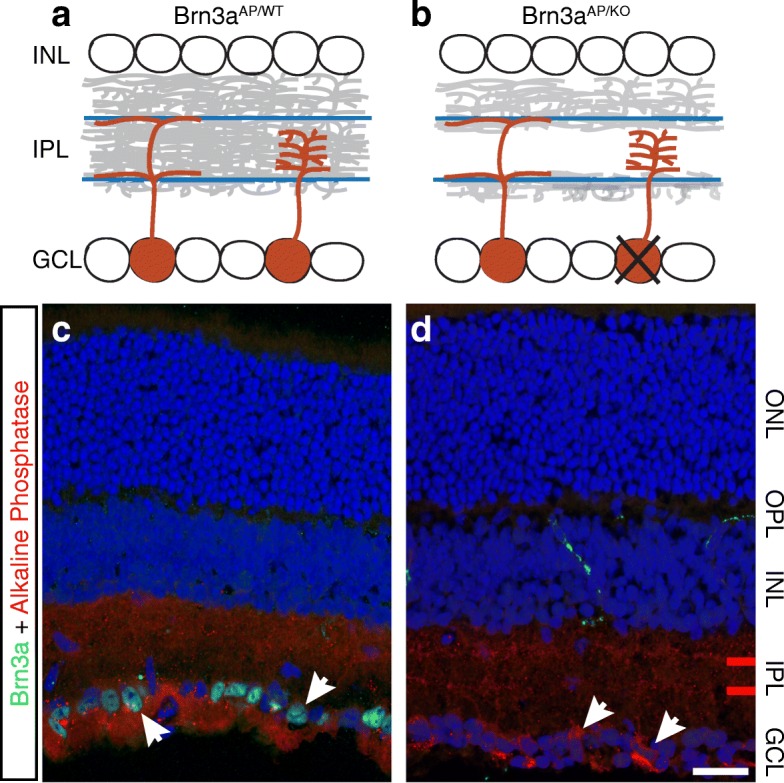
Retinal Brn3a ablation results in specific RGC defects. **a-b** Summary of Brn3a RGC phenotype, based on previous work [2, 9, 28]. Distribution of dendritic arbors for Brn3a heterozygote (**a**, Brn3a^AP/WT^) and Brn3a knock-out (**b**, Brn3a^AP/KO^) RGCs. **a** Dendrites of Brn3a^AP/WT^ RGCs, including ON-OFF-DS bistratified (left) and midget-like (right) cells, occupy the outer two-thirds of the Inner Plexiform Layer (IPL, gray). **b** Midget-like cells are absent from Brn3a^AP/KO^ RGCs, and the dendrites of remaining cells are co-stratifying with the inner and outer Starburst Amacrine cell dendritic arbors (blue lines). **c-d** Indirect immunofluorescence illustrating Brn3a^AP^ RGC arbors and cell bodies (red), and Brn3a transcription factor expression (green) in Rax:Cre; *Brn3a*^*CKOAP/WT*^ (**c**) and Rax:Cre; *Brn3a*^*CKOAP/KO*^ (**d**) adult retinas. Note the gap of Brn3a^AP^ staining in **d**, and the absence of Brn3a TF positive cell bodies in the GCL. ONL = Outer Nuclear Layer; OPL = Outer Plexiform Layer; INL = Inner Nuclear Layer; IPL = Inner Plexiform Layer; GCL = Ganglion Cell Layer. Red bars in IPL of (**d**) indicate the position of inner and outer ChAT bands. Arrowheads are showing AP-expressing cells. Scale bar in D, 25 μm

In order to achieve complete ablation of *Brn3a* from RGCs, we crossed females homozygote for the previously reported *Brn3a*^*CKOAP/CKOAP*^ allele [9] with Rax:Cre; *Brn3a*^*KO/WT*^ males. Rax:Cre is a BAC transgenic allele [49] that provides essentially complete Cre recombination beginning at early stages of eye development (Embryonic day 9, E9). Offspring were either Rax:Cre; *Brn3a*^*CKOAP/WT*^ resulting in Brn3a heterozygote RGCs (henceforth Brn3a^AP/WT^ RGCs) or Rax:Cre; *Brn3a*^*CKOAP/KO*^ resulting in Brn3a knockout RGCs (henceforth Brn3a^AP/KO^ RGCs). As previously reported, Brn3a protein is expressed in a large fraction of Ganglion Cell Layer (GCL) nuclei, and dendrites of Brn3a^AP/WT^ RGCs occupy the outer two-thirds of the Inner Plexiform Layer (IPL, Fig. 1a, c). However, in Rax:Cre; *Brn3a*^*CKOAP/KO*^ retinas there are essentially no Brn3a positive cells, and the dendrites of Brn3a^AP/KO^ RGCs show a gap in AP-positive lamination (Fig. 1b, d). This gap, which extends between the characteristic positions of Choline-Acetyl-Transferase (ChAT) positive Starburst Amacrine cells, is largely due to loss of RGCs with small and dense dendritic arbor (midget-like cells) (Fig. 1b, d) [2, 9, 28]. In order to capture the essential events in RGC type specification (dendrite formation, axon invasion into retinorecipient nuclei, synaptogenesis, and functional maturation) we prepared retinal sections from Rax:Cre; *Brn3a*^*CKOAP/KO*^ and Rax:Cre; *Brn3a*^*CKOAP/WT*^ littermate controls at five postnatal ages – P0, P3, P7, P14 and P22.

The genes screened in this study represent a subset of potential Brn3a target genes identified by comparing transcript level RNASeq data from Brn3a^AP/KO^ and Brn3a^AP/WT^ postnatal day 3 RGCs [5]. Genes selected in our ISH analysis had to have at least one transcript with more than 2 FPKM (fragments per million reads per gene kilobase) expression level in RGCs, and at least a two-fold differential in Brn3a^AP/WT^ versus Brn3a^AP/KO^ RGCs (Brn3a regulated or dependent). In addition, the Brn3a dependent transcript should show no Brn3b regulation (less than two-fold differential in P3 Brn3b^AP/WT^ versus Brn3b^AP/KO^ RGCs). From the set of 180 differentially expressed transcripts that had these criteria (identified in [5]), we focused on genes whose products had potential functional association with gene regulation, synapse formation, cell adhesion, vesicle transport, cytoskeleton changes, intracellular signaling, or secreted molecules. These molecular classes should be particularly important during RGC type specification.

**Fig. 2:**
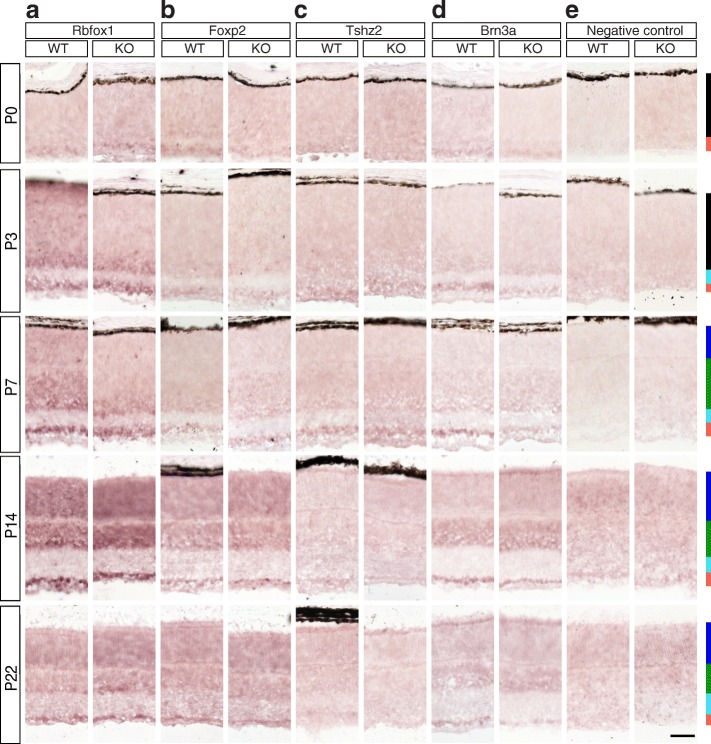
Candidate Brn3a target genes: Transcriptional and Translational regulators. In situ hybridization profiles for potential Brn3a target genes. Retinal sections from Rax:Cre; *Brn3a*^*CKOAP/WT*^ (left panel, WT) and Rax:Cre; *Brn3a*^*CKOAP/KO*^ (right panel, KO) mice, harvested at Postnatal days 0, 3, 7, 14 and 22 (P0, P3, P7, P14 and P22). The in situ hybridization probes were generated against the 3’-UTR of the corresponding target gene, using primers indicated in Table 1. A positive control (**d**, Brn3a) and a negative control (**e**, no probe) are shown. Bars on the right represent retina layers positions: black – NBL (neuroblast layer), red – GCL, cyan – IPL, blue – ONL, green – INL. **a** Rbfox1, **b** Foxp2, **c** Tshz2. Scale bar in (**e**), 50 μm

**Fig. 3:**
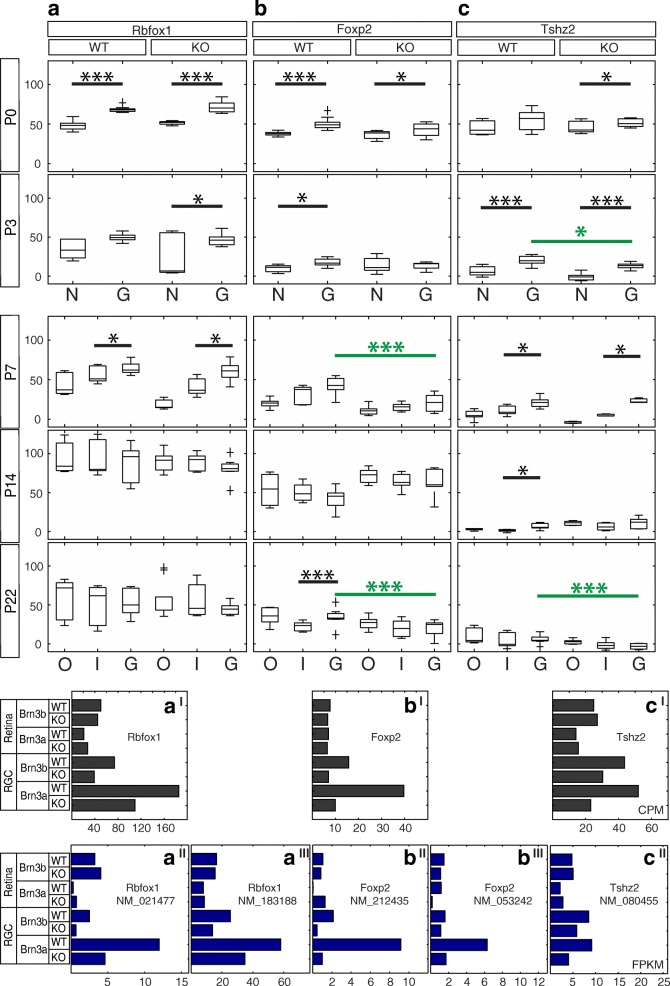
Candidate Brn3a target genes: Transcriptional and Translational regulators. RNASeq and ISH quantitation**.** In situ hybridization quantitation (**a-c**), and gene (*a*^*I*^*- c*^*I*^) and transcript (*a*^*II*^*- c*^*II*^) level RNASeq profiles for potential Brn3a target genes. **a-c** Box-whiskers plots for NBL and GCL of P0 and P3, and ONL, INL and GCL for P7-P22 retinas from Rax:Cre; *Brn3a*^*CKOAP/WT*^ (left panel, WT) and Rax:Cre; *Brn3a*^*CKOAP/KO*^ (right panel, KO) mice, harvested at P0, P3, P7, P14 and P22 show normalized (all except P0) mean intensity values from images of retinal sections (Y axis). Individual values for each layer are normalized to the respective IPL value in P3-P22 cases. X axis represents retinal layers: N – NBL, G – GCL, O – ONL, I – INL. Horizontal bars in panels denote observation pairs showing significant expression differences (Kolmogorov-Smirnov - KS2 test) between INL/NBL and GCL (black bar) and Brn3a-dependency by comparing respective WT and KO GCL values (green bar; significance levels * *p* < 0.05, ** *p* < 0.01, *** *p* < 0.001). All values and KS2 test outcomes are provided in Additional file 1: Table S1. *a*^*I*^*-c*^*I*^ Gene level RNASeq profiles from affinity purified Brn3^AP^ RGCs (RGC) and retinal supernatants (Retina) derived from P3 mice with the following genotypes: Pax6α:Cre; *Brn3a*^*CKOAP/WT*^ (Brn3a-WT), Pax6α:Cre; *Brn3a*^*CKOAP/KO*^ (Brn3a-KO), Pax6α:Cre; *Brn3b*^*CKOAP/WT*^ (Brn3b-WT), and Pax6α:Cre; *Brn3b*^*CKOAP/KO*^ (Brn3b-KO) (Sajgo et al. 2017). Values on the x axis are in CPM (counts per million reads), and bars represent mean values for two replicates (RGC samples) and single samples (retina supernatants). *a*^*II*^*-c*^*II*^ Transcript level RNASeq profiles from the same samples as in *a*^*I*^*-c*^*I*^ Values on the x axis are in FPKM, and bars represent mean values for two replicates (RGC samples) and single samples (retina supernatants). For each gene, only transcripts having detectable (> 1 FPKM values) in at least one of the samples are presented. The transcript (NM) number is indicated under the gene name. *a*^*I*^*-a*^*III*^ Rbfox1, *b*^*I*^*-b*^*III*^ Foxp2, *c*^*I*^*-c*^*II*^ Tshz2

**Fig. 4:**
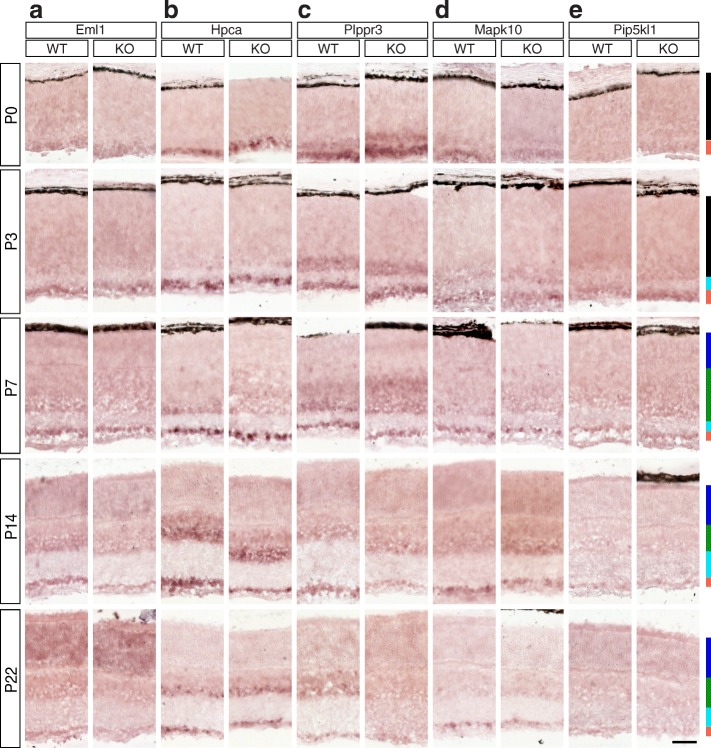
Candidate Brn3a target genes: Intracellular signaling and cytoskeleton-associated proteins. **a-e** In situ hybridization analysis in WT and Brn3a-KO mouse retinas at 5 postnatal ages. **a** Eml1, **b** Hpca, **c** Plppr3, **d** Mapk10, **e** Pip5kl1. All samples were collected, imaged and formatted as in Fig. 2. Scale bar – 50 μm

**Fig. 5:**
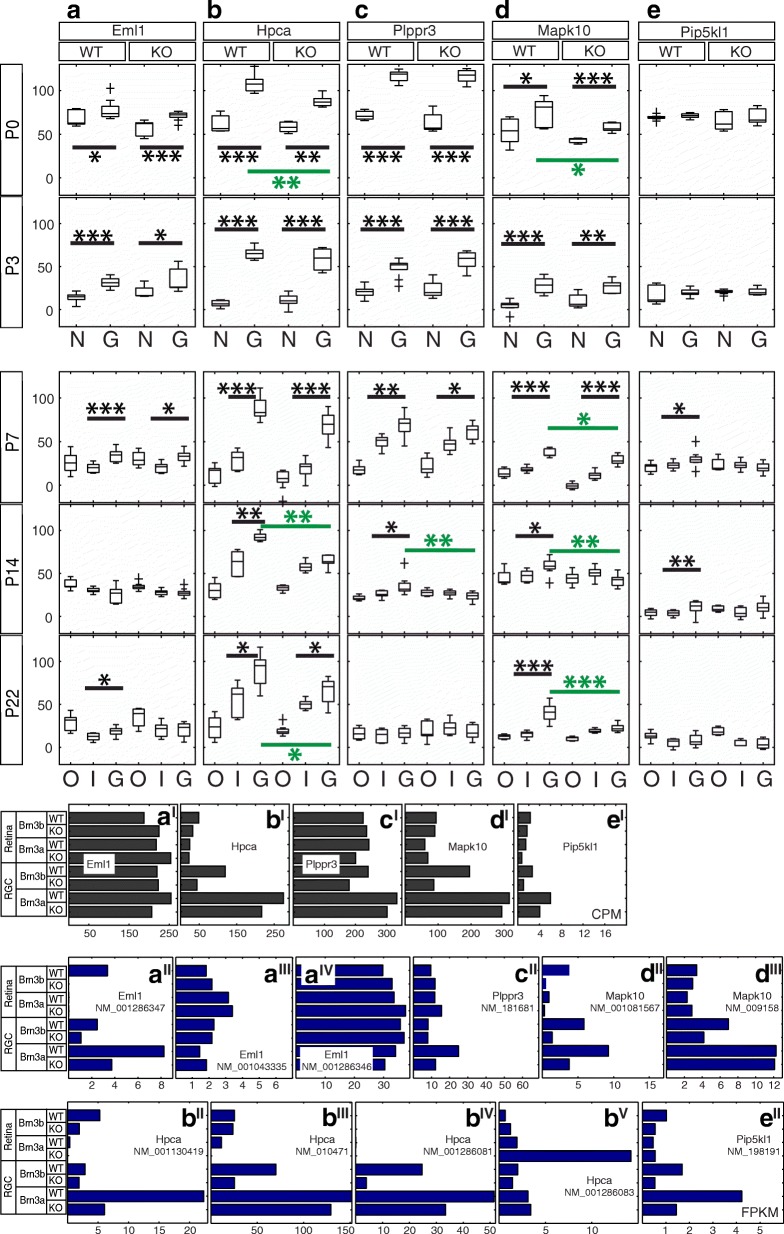
Candidate Brn3a target genes: Intracellular signaling and cytoskeleton-associated proteins. RNASeq and ISH quantitation. **a-e** In situ hybridization quantitation analysis in WT and Brn3a-KO mouse retinas at 5 postnatal ages. *a*^*I*^*-e*^*I*^ RNASeq gene level profiles at P3 (expressed in CPM). *a*^*II*^*-e*^*II*^ RNASeq transcript level profiles expressed in FPKM for all transcripts of the five genes detected at more than 1 FPKM in at least one P3 retinal sample. *a-a*^*IV*^ Eml1, *b-b*^*V*^ Hpca, *c-c*^*II*^ Plppr3, *d-d*^*III*^ Mapk10, *e-e*^*II*^ Pip5kl1. Plot formatting and annotations as in Fig. 3

**Fig. 6:**
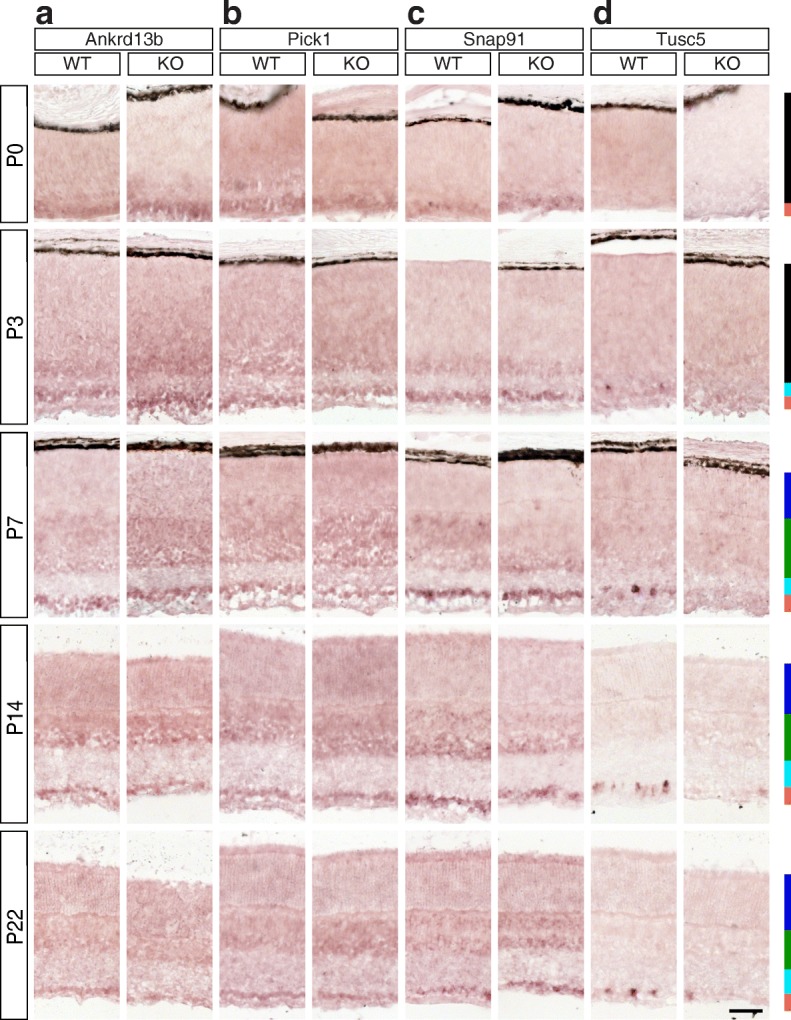
Candidate Brn3a target genes: Vesicle-associated proteins. **a-d** In situ hybridization analysis in WT and Brn3a-KO mouse retinas at 5 postnatal ages. **a** Ankrd13b, **b** Pick1, **c** Snap91, **d** Tusc5. All samples were collected, imaged and formatted as in Fig. 2. Scale bar – 50 μm

**Fig. 7:**
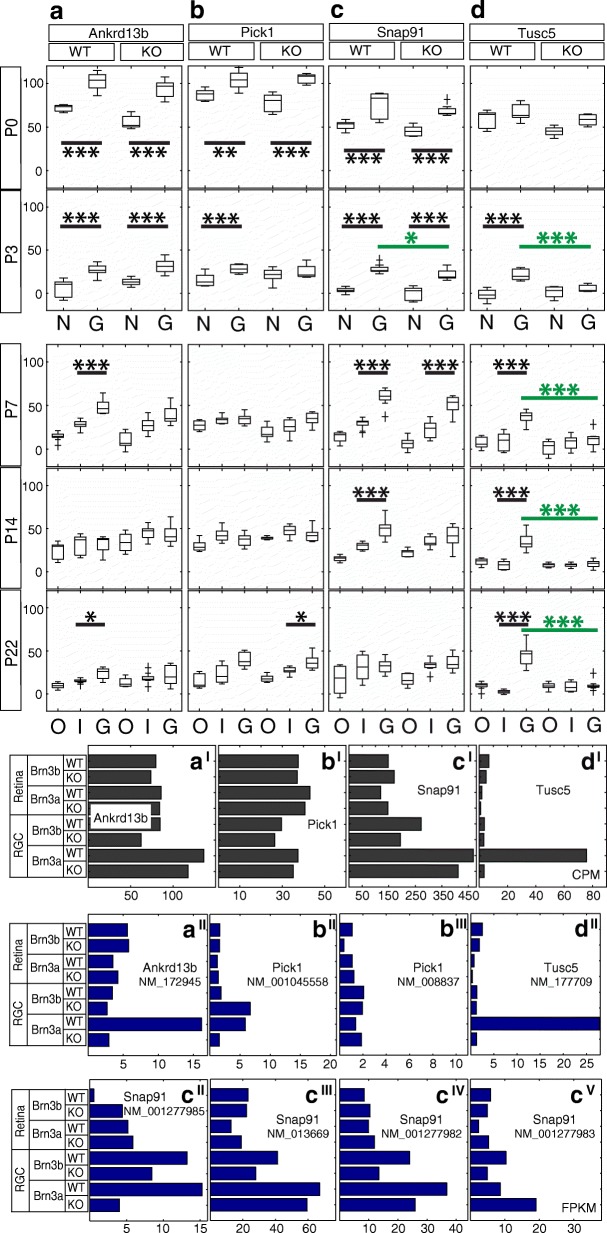
Candidate Brn3a target genes: Vesicle-associated proteins. RNASeq and ISH quantitation. **a-d** In situ hybridization quantitation analysis in WT and Brn3a-KO mouse retinas at 5 postnatal ages. *a*^*I*^*-d*^*I*^ RNASeq gene level profiles at P3 (expressed in CPM). *a*^*II*^*-d*^*II*^ RNASeq transcript level profiles expressed in FPKM for all transcripts of the four genes detected at more than 1 FPKM in at least one P3 retinal sample. *a-a*^*II*^ Ankrd13b, *b-b*^*III*^ Pick1, *c-c*^*V*^ Snap91, *d-d*^*II*^ Tusc5. Plot formatting and annotations as in Fig. 3

**Fig. 8:**
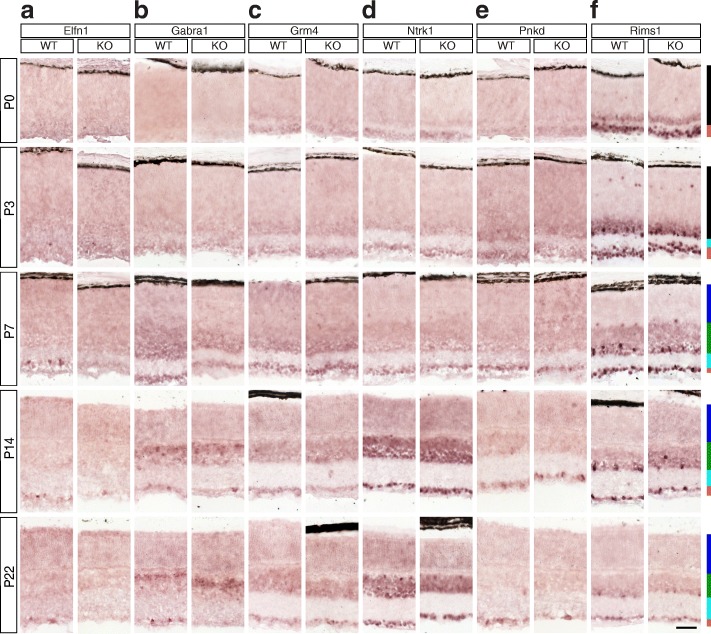
Candidate Brn3a target genes: Synapse-associated proteins. **a-f** In situ hybridization analysis in WT and Brn3a-KO mouse retinas at 5 postnatal ages. **a** Elfn1, **b** Gabra1, **c** Grm4, **d** Ntrk1, **e** Pnkd, **f** Rims1. All samples were collected, imaged and formatted as in Fig. 2. Scale bar – 50 μm

**Fig. 9:**
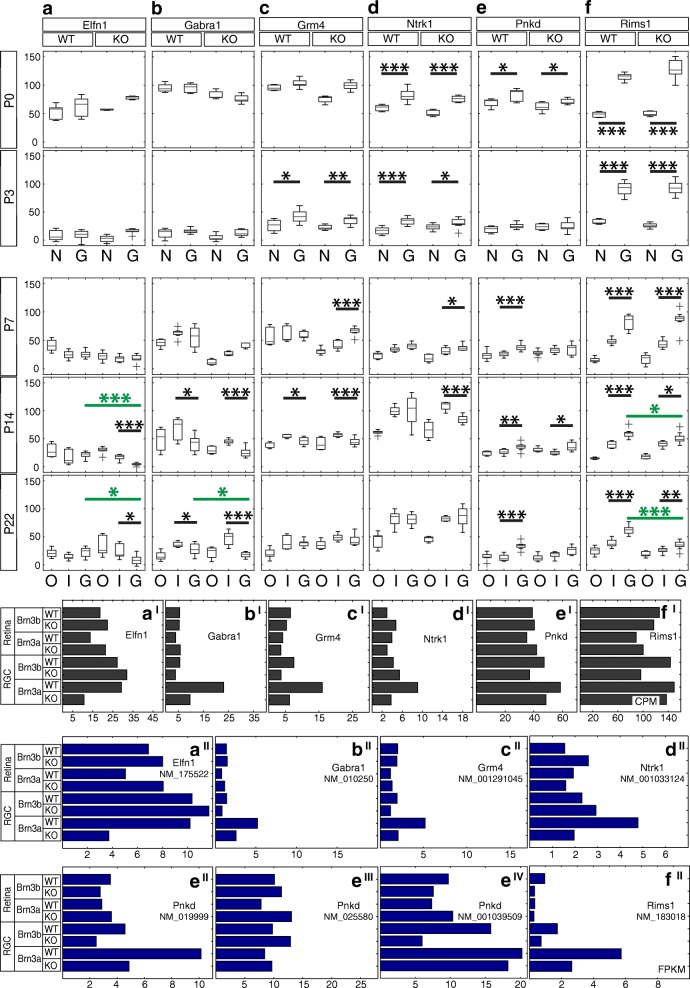
Candidate Brn3a target genes: Synapse-associated proteins. RNASeq and ISH quantitation. **a-f** In situ hybridization quantitation analysis in WT and Brn3a-KO mouse retinas at 5 postnatal ages. *a*^*I*^*-f*^*I*^ RNASeq gene level profiles at P3 (expressed in CPM). *a*^*II*^*-f*^*II*^ RNASeq transcript level profiles expressed in FPKM for all transcripts of the six genes detected at more than 1 FPKM in at least one P3 retinal sample. *a-a*^*II*^ Elfn1, *b-b*^*II*^ Gabra1, *c-c*^*II*^ Grm4, *d-d*^*II*^ Ntrk1, *e-e*^*IV*^ Pnkd, *f-f*^*II*^ Rims1. Plot formatting and annotations as in Fig. 3

**Fig. 10:**
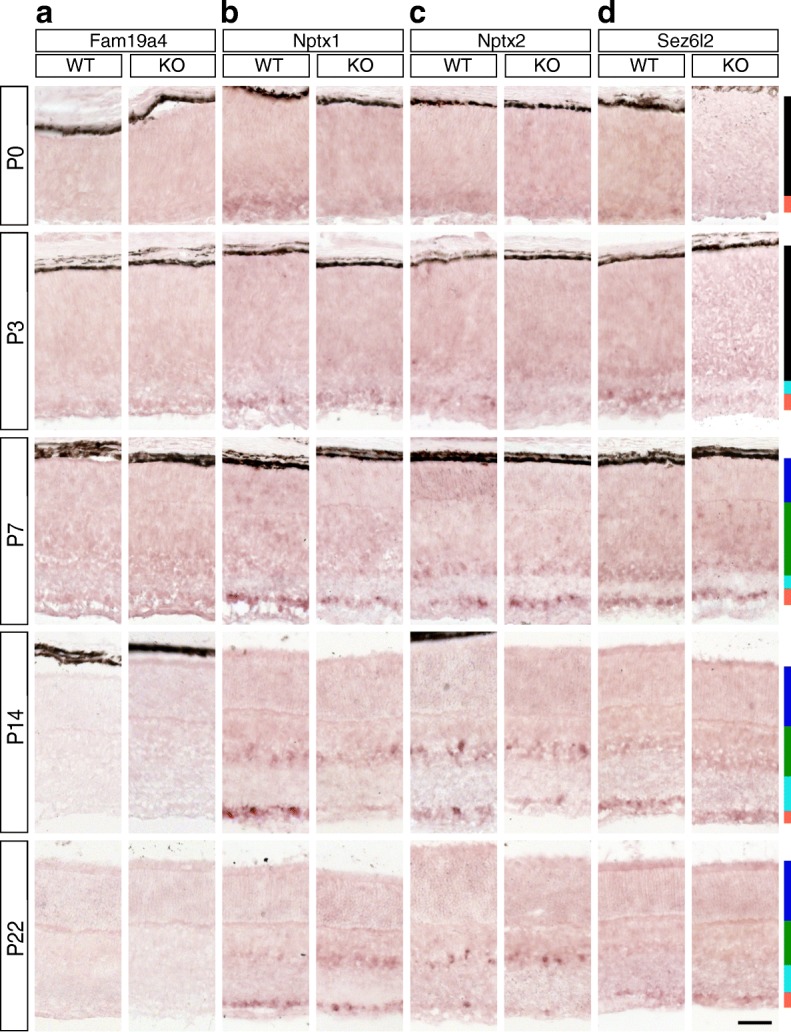
Candidate Brn3a target genes: Secreted proteins. **a-d** In situ hybridization analysis in WT and Brn3a-KO mouse retinas at 5 postnatal ages. **a** – Fam19a4, **b** – Nptx1, **c** – Nptx2, **d** – Sez6l2. All samples were collected, imaged and formatted as in Fig. 2. Scale bar – 50 μm

**Fig. 11:**
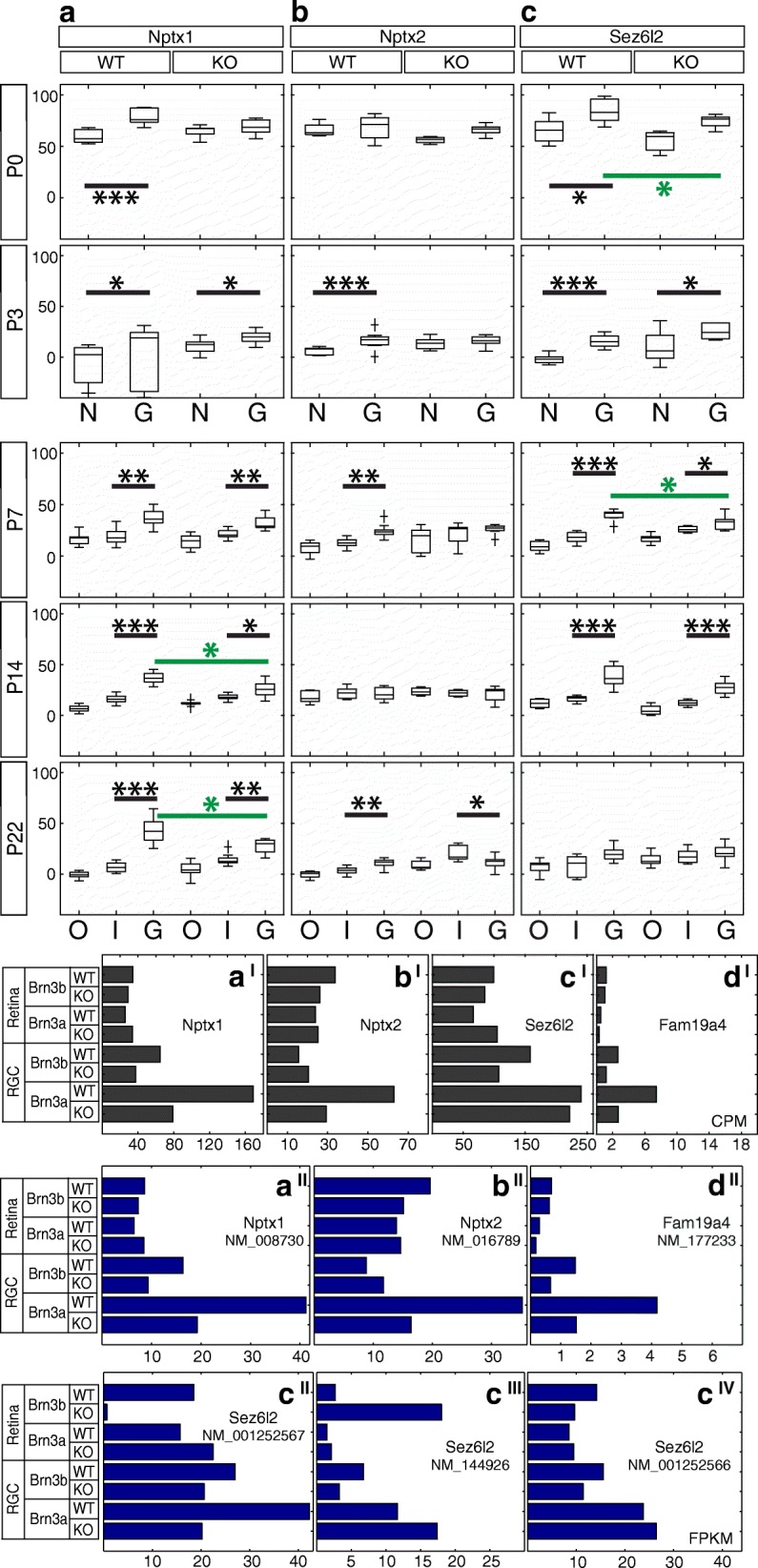
Candidate Brn3a target genes: Secreted proteins. RNASeq and ISH quantitation. **a-c** In situ hybridization quantitation analysis in WT and Brn3a-KO mouse retinas at 5 postnatal ages. *a*^*I*^*-d*^*I*^ RNASeq gene level profiles at P3 (expressed in CPM). *a*^*II*^*-d*^*II*^ RNASeq transcript level profiles expressed in FPKM for all transcripts of the four genes detected at more than 1 FPKM in at least one P3 retinal sample. *a-a*^*II*^ Nptx1, *b-b*^*II*^ Nptx2, *c-c*^*IV*^ Sez6l2, *d*^*I*^*-d*^*II*^ Fam19a4. Plot formatting and annotations as in Fig. 3.

**Fig. 12:**
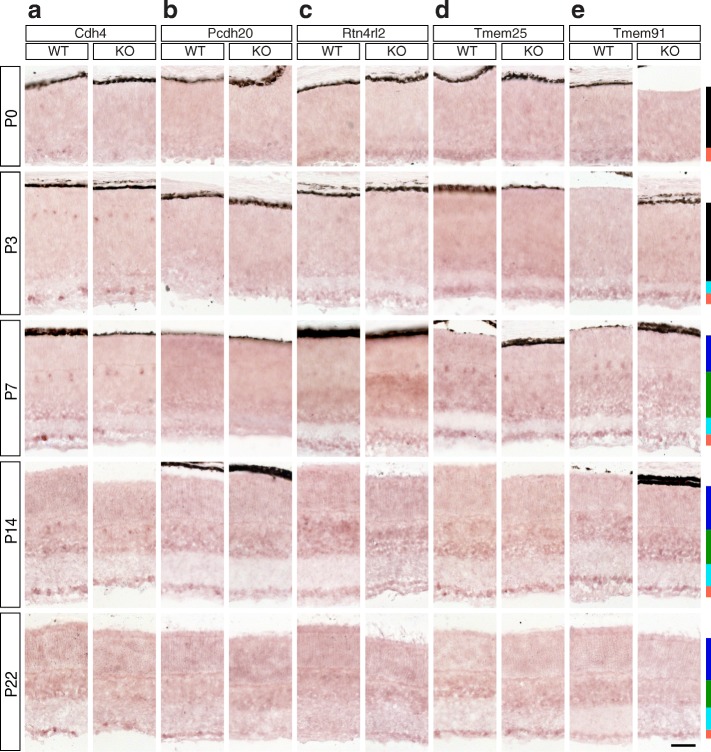
Candidate Brn3a target genes: Adhesion molecules and other transmembrane proteins. **a-e** In situ hybridization analysis in WT and Brn3a-KO mouse retinas at 5 postnatal ages. **a** Cdh4, **b** Pcdh20, **c** Rtn4rl2, **d** Tmem25, **e** Tmem91. All samples were collected, imaged and formatted as in Fig. 2. Scale bar – 50 μm.

**Fig. 13:**
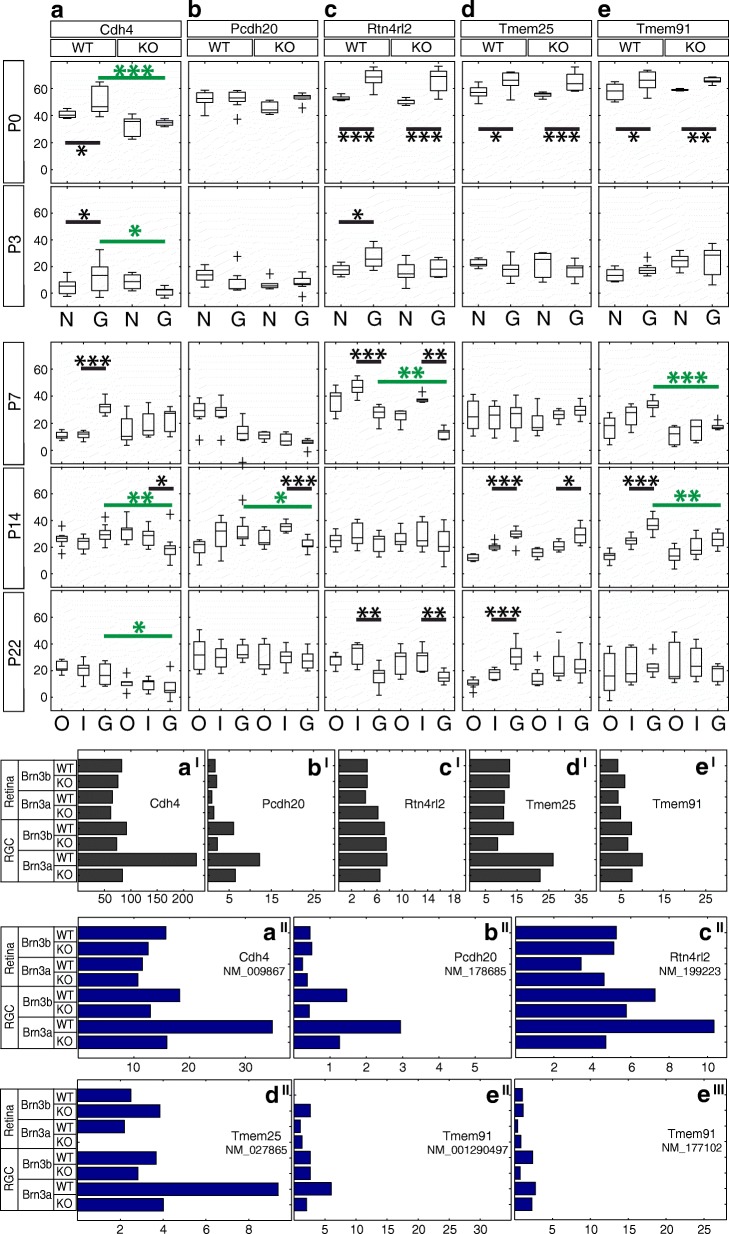
Candidate Brn3a target genes: Adhesion molecules and other transmembrane proteins. RNASeq and ISH quantitation. **a-e** In situ hybridization quantitation analysis in WT and Brn3a-KO mouse retinas at 5 postnatal ages. *a*^*I*^*-e*^*I*^ RNASeq gene level profiles at P3 (expressed in CPM). *a*^*II*^*-e*^*III*^ RNASeq transcript level profiles expressed in FPKM for all transcripts of the five genes detected at more than 1 FPKM in at least one P3 retinal sample. *a-a*^*II*^ Cdh4, *b-b*^*II*^ Pcdh20, *c-c*^*II*^ Rtn4rl2, *d-d*^*II*^ Tmem25, *e-e*^*III*^ Tmem91. Plot formatting and annotations as in Fig. 3

In Figs. 2, 3, 4, 5, 6, 7, 8, 9, 10, 11, 12 and 13, we provide RNASeq data for transcript and gene expression and ISH results together with its quantitation for all target genes, organized by molecular class. ISH quantitation results are summarized in Additional file 1: Table S1. RNASeq data for transcript expression are derived from the Sajgo et al. [5] data set and gene level analysis is based on the same dataset (however it takes into account all possible transcript isoforms, not only RefSeq ones). RNASeq data for transcript expression represent all RefSeq transcripts that had at least 1 FPKM expression level in at least one retinal or RGC sample and including the ones that passed the above selection criteria. Note that the x scales for each plot are different, depending on the range of FPKM/CPM (counts per million reads) values in the individual samples. The ISH panels show the gene level analysis, with riboprobes directed against the 3’-Untranslated Regions (3’-UTR), which are shared by all transcripts.

### Transcriptional and translational regulators (Figures 2 and 3)

Two transcription factors (Foxp2 and Tshz2) and one RNA processing factor (Rbfox1) passed our selection criteria. Two Rbfox1 (RNA binding protein, fox-1 homolog 1) RefSeq transcripts were significantly expressed in P3 Brn3a^+^ RGCs compared to the retina (NM_021477 and NM_183188, Fig. 3a^II^-a^III^), but only NM_021477 showed significant Brn3a dependence. Gene level RNASeq analysis for Rbfox1 also revealed both RGC-enrichment as well as tendency towards Brn3a-mediated regulation of expression (Fig. 3a^I^). The in situ time series (Fig. 2a) confirmed relative GCL enrichment for Rbfox1 from P0-P7 (P0 WT and KO – *p* < 0.001; P3 KO, P7 WT and KO – *p* < 0.05, Additional file 1: Table S1 A-C), however beginning with P7, the INL and eventually the ONL turned positive (Figs. 2a, 3a). Differential expression between Brn3aWT and KO retinas was not observed. Both Foxp2 (forkhead box P2) transcripts expressed in the retina (NM_212435 and NM_053242) were selectively enriched in Brn3a RGCs and appeared regulated by Brn3a (Fig. 3b^II^-b^III^). Gene level RNASeq analysis also showed strong differential expression in Brn3a WT RGCs compared to Brn3a KO RGCs. Enrichment of Foxp2 expression in the GCL was confirmed by ISH in early time-points (P0-P3) and in the adult, while the significant (*p* < 0.001) regulation by Brn3a in the GCL was observed at P7 and P22 (Figs. 2b, 3b, Additional file 1: Table S1). Tshz2 (teashirt zinc finger homeobox 2) showed enrichment in RGCs vs. retina as well as Brn3a dependency at both gene and transcript level (Fig. 3c^I^-c^II^). ISH confirms modest but significant GCL enrichment from P0-P14 and Brn3a regulation at P3 (*p* < 0.05) and P22 (*p* < 0.001, Figs. 2c, 3c, Additional file 1: Table S1). Note that Brn3a signal can still be detected in the GCL of Rax:Cre; *Brn3a*^*CKOAP/KO*^ (KO) retinas (Fig. 2d), since the ISH probe is directed against the 3’UTR region which is not ablated in the recombined conditional knock-in allele (Brn3a^AP^) and Brn3a ablation results in only 30–40% reduction in total Brn3a^+^ RGC numbers [5, 9, 28].

### Intracellular signaling and cytoskeleton-associated proteins (Figures 4 and 5)

Eml1 (echinoderm microtubule associated protein like 1) has 3 transcripts (NM_001286347, NM_001043335 and NM_001286346, Fig. 5a^II^-a^IV^), and only one of them (NM_001286347) shows Brn3a RGC enrichment and Brn3a dependence. Gene level analysis shows even distribution of expression between RGC and retina samples at P3 (Fig. 5a^I^). ISH (Fig. 4a) demonstrates that Eml1 is significantly GCL enriched in both WT and Brn3a KO from P0-P7, however beginning with P14, the INL and later the ONL become positive (Fig. 5a, Additional file 1: Table S1). Eml1 is not differentially expressed between Brn3a WT and KO retinas throughout studied postnatal developmental period. Hpca (hippocalcin) has 4 transcripts expressed in the retina (NM_001130419, NM_010471, NM_001286081 and NM_001286083). First three of them were highly expressed in P3 Brn3a^AP^ RGCs compared to the retina (Fig. 5b^II^-b^IV^), while NM_001286083 (Fig. 5b^V^) was selectively enriched in Brn3a^KO^ retina. Only NM_001130419 appeared regulated by Brn3a (Fig. 5b^II^). Gene level analysis revealed enrichment in both Brn3aWT and Brn3aKO RGCs (Fig. 5b^I^). Strong and significant GCL enrichment was confirmed by ISH from P0-P7, however beginning with P14 Hpca is highly expressed in both GCL and inner INL. A modest reduction in Hpca level is apparent in the GCL of Brn3a^KO^ retinas at P0 (*p* < 0.01), P14 (*p* < 0.01) and P22 (*p* < 0.05, Figs. 4b, 5b, Additional file 1: Table S1). The only expressed Plppr3 (phospholipid phosphatase related 3) transcript shows a moderate enrichment in P3 RGCs vs. retina (Fig. 5c^II^), while RNASeq gene level analysis does not reveal any strong signs of GCL enrichment or Brn3a dependency (Fig. 5c^I^). ISH time series confirms the transcript RNASeq results in terms of GCL enrichment from P0-P14 (Figs. 4c, 5c, Additional file 1: Table S1 A-D). The expression of Plppr3 decreases towards adult and this gene is not affected by Brn3a loss at P0-P7, while at P14 we found significant differential expression between Brn3a WT and KO (*p* < 0.01, Fig. 5c, Additional file 1: Table S1 A-D). Both Mapk10 (mitogen-activated protein kinase 10) isoforms (NM_001081567 and NM_009158, Fig. 5d^II^-d^III^) are enriched in RGCs compared to the retina. Only one of them (NM_001081567) appears to be regulated by Brn3a (and also Brn3b) at P3. Gene level analysis confirms only enrichment in Brn3a WT and KO samples, and differential expression between Brn3b WT and KO (Fig. 5d^I^). ISH reveals significant Mapk10 GCL enrichment throughout postnatal development (P0-P22, Figs. 4d, 5d, Additional file 1: Table S1). Beginning with P7, Mapk10^+^ cell bodies sparsely label the GCL, and their number appears to be reduced in Brn3a^KO^ retinas compared to the WT at the majority of studied ages (P0 *p* < 0.05, P7 *p* < 0.05, P14 *p* < 0.01, P22 *p* < 0.001, Additional file 1: Table S1). Transcript and gene RNASeq profiles show enrichment, and in case of a transcript – Brn3a dependency of Pip5kl1 (phosphatidylinositol-4-phoshate 5-kinase like 1) expression in P3 RGCs vs. retina (Fig. 3e^I^-e^II^). However, in situ time series confirms GCL enrichment only at P7 (*p* < 0.05) and P14 (*p* < 0.01), and it does not reveal a considerable Brn3a regulation of Pip5kl1 (Figs. 4e, 5e, Additional file 1: Table S1).

### Vesicle-associated proteins (Figures 6 and 7)

Transcript level RNASeq shows that the only expressed Ankrd13b (ankyrin repeat domain 13b) RefSeq transcript is strongly enriched in Brn3a^AP/WT^ RGCs compared to the retina and other RGC samples (Fig. 7a^II^). At the same time, gene level analysis revealed only the enrichment in Brn3a WT and KO samples but not the dependency on Brn3a (Fig. 7a^I^). ISH confirms strong enrichment in the GCL from P0-P7, which decreases towards adult (Fig. 7a). There is no significant Brn3a regulation in the GCL at any of the studied ages (Additional file 1: Table S1). Two Pick1 (protein interacting with C kinase 1) transcripts (NM_001045558 and NM_008837, Fig. 7b^II^-b^III^) demonstrated a modest enrichment in RGCs vs. retina at P3, while gene expression RNASeq analysis did not show any signs of it (Fig. 7b^I^). One of the transcripts (NM_001045558) was enriched in Brn3a^AP/WT^ and Brn3b^AP/KO^ RGCs, suggesting positive regulation by Brn3a and negative regulation by Brn3b (Fig. 7b^II^). ISH confirms GCL enrichment from P0-P3, however it does not show a significant Brn3a-dependent differential expression (Figs. 6b and 7b, Additional file 1: Table S1). Snap91 (synaptosome associated protein 91) gene has 4 retinally expressed isoforms (NM_001277985, NM_013669, NM_001277982 and NM_001277983, Fig. 7c^II^-c^V^), all of which showed enrichment in RGCs compared to the retina. However only NM_001277985 shows a strong dependency on Brn3a expression in RGCs (Fig. 7c^II^). Gene level analysis confirms RGC enrichment, especially in Brn3a RGC samples, but it does not show Brn3a-dependent differential expression (Fig. 7c^I^). ISH time series confirms a significant enrichment in the GCL from P0-P14, and shows the only time point (P3) with significant (*p* < 0.05) Brn3a dependency of expression (Figs. 6c, 7c, Additional file 1: Table S1). According to P3 RNASeq data (Fig. 7d^I^-d^II)^, Tusc5 (tumor suppressor candidate 5) is highly enriched in Brn3a^AP/WT^ RGCs, compared to the retina and other RGC samples, and strongly regulated by Brn3a. ISH reveals intensely stained sparse cell bodies in the GCL of Brn3a WT retinas, which are nearly completely lost in Brn3a^KO^ (Fig. 6d). ISH quantitation results confirm strong GCL enrichment in the Brn3a WT retinas, and almost absolute dependency of GCL expression on Brn3a (Fig. 7d, Additional file 1: Table S1).

### Synapse-associated proteins (Figures 8 and 9)

**Fig. 14:**
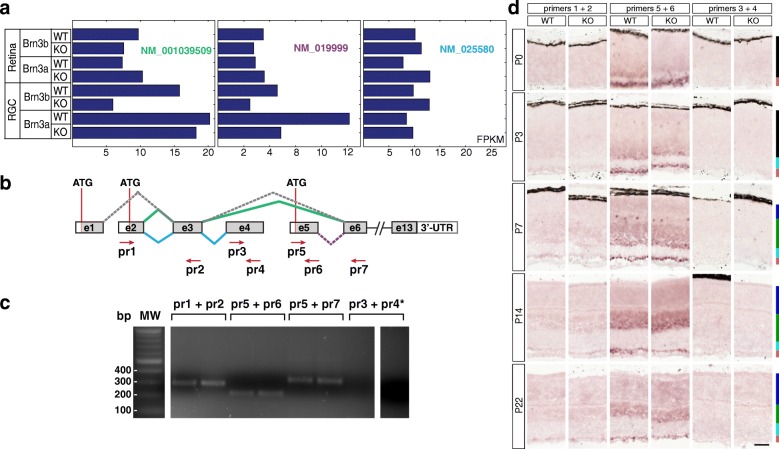
Pnkd transcripts detected in the retina. **a** RNAseq profiles expressed in FPKM of three expressed Pnkd RefSeq transcripts from retinal and RGC-derived samples of Brn3a and Brn3b WT and KO mice at P3. **b** Predicted Pnkd splicing variants. Exons are represented as boxes and numbered. White boxes are untranslated regions, gray boxes are coding regions. Splicing sequences for alternative transcripts are indicated above or below the exons. Green lines indicate connected exons for transcript NM_001039509, blue – for NM_025580, purple – for NM_019999. Gray lines represent a putative transcript (XM_006496167) that was not detected by RNAseq. Exons 6–13 are common to NM_001039509 and NM_019999. Red arrows show the positions of primers made to check the presence of each predicted variant (see also Table 2). **c** Agarose-gel electrophoresis showing RT-PCR reactions from P3 retina RNA. Indicated primer pairs refer to B. Duplicates are shown for each primer combination. The expected sizes for all primer combinations are indicated in Table 2. All bands generated by RT-PCR were gel-extracted, subcloned and sequenced for confirmation. **d** in situ hybridization analysis in WT and Brn3a-KO mouse retinas at 5 postnatal ages. RNA probes were designed to detect transcript-specific exons: primers 1 and 2 for NM_001039509 (green) and NM_025580 (blue) isoforms, primers 5 and 6 specifically for NM_019999 (purple), and primer 3 and 4 –specifically for NM_025580. Scale bar – 50 μm

A single expressed Elfn1 (leucine rich repeat and fibronectin type III, extracellular 1) transcript is expressed in both retina and RGCs, and RNASeq demonstrated its profound differential between Brn3a^AP/WT^ and Brn3a^AP/KO^ RGCs (Fig. 9a^II^), and gene level analysis confirms this result (Fig. 9a^I^). ISH reveals intense sparse labelling in the GCL beginning with P3, peaking around P7 but persisting in the adult (Fig. 8a). Due to the sparseness of expression the quantitation does not allow to capture significant GCL enrichment (Fig. 9a, Additional file 1: Table S1). Starting at P14, Elfn1 expression can also be seen in the INL and ONL. Beginning with P7, the number of intensely labelled GCL cell bodies declines in the Brn3a KO retinas (Fig. 8a), and this reduction reaches significant levels at P14 (*p* < 0.001) and persists in adult stage (*p* < 0.05, Fig. 9a, Additional file 1: Table S1 D-E). Surprisingly, due to downregulation in Brn3a KO GCL, we also can see a significant enrichment of expression in the INL compared to the GCL at P14 (*p* < 0.001) and adult age (*p* < 0.05). Gabra1 (gamma-aminobutyric acid A receptor) has one transcript that is enriched in Brn3a^AP/WT^ RGCs compared to the retina and downregulated in Brn3a^AP/KO^ RGCs (Fig. 9b^II^), which is confirmed by gene level RNASeq analysis (Fig. 9b^I^). ISH reveals the start of the expression at P3 in the GCL and P7 in the INL with progressive increase of the signal towards P14 and P22 (Fig. 8b). There are sparse intensely labelled cells in both INL and GCL of P14 and P22 Brn3a WT retina. And due to the sparseness of expression in the GCL but not as much in the INL, the INL levels are significantly higher compared to GCL level in both Brn3a WT and KO at P14 and P22 (Fig. 9b, Additional file 1: Table S1 D-E). Moreover, P22 Brn3a KO retinas lack positive signal in the GCL but not the INL, suggesting a strong Brn3a dependency of Gabra1 in RGCs but not INL cells (likely Bipolar and/or Amacrine cells). Both transcript and gene level RNASeq analysis suggests that Grm4 (glutamate metabotropic receptor 4) – is enriched in Brn3a^AP/WT^ RGCs and regulated by Brn3a (Fig. 9c^I^-c^II^). ISH reveals Grm4 expression in both GCL and INL (NBL) beginning with P0-P7 and continuing towards adult with no visible downregulation in Brn3a KO retinas (Fig. 8c). ISH quantitation shows a significant level of GCL enrichment at P3 (WT *p* < 0.05, KO < 0.01), which later reverts into INL enrichment (P14 WT *p* < 0.05, KO < 0.001, Fig. 9c). ISH results do not support dependency of Grm4 expression on Brn3a (Additional file 1: Table S1). Ntrk1 (neurotrophic tyrosine kinase, receptor, type 1) is expressed both in retina and RGCs and differentially regulated by Brn3a (Fig. 9d^I^-d^II^). ISH confirms significant Ntrk1 GCL enrichment at P0 and P3 (Figs. 8d, 9d, Additional file 1: Table S1 A-B). The INL expression starts at P3, catches up with the GCL at P7-P14, and they both proceed into adult age. Ntrk1 is expressed exceptionally highly at P14 and P22, however no differential between Brn3a WT and KO retinas is apparent at any age (Figs. 8d, 9d, Additional file 1: Table S1). Pnkd (paroxysmal non-kinesigenic dyskinesia) has three RefSeq isoforms (NM_019999, NM_025580 and NM_001039509, Fig. 9e^II^-e^IV^), all of them seem to be expressed in both RGCs and retina, but only one of them is regulated by Brn3a (NM_019999, Fig. 9e^II^). Gene level analysis demonstrates broad expression in both RGCs and retina with no apparent regulation by Brn3a (Fig. 9e^I^). The 3’-UTR in situ probe common for all 3 isoforms allows to find Pnkd expression in the GCL and other retinal layers beginning with P0-P3 (Fig. 8e). We find significant enrichment of Pnkd expression in the GCL of Brn3a WT retinas at most of studied ages (P0 *p* < 0.05, P7 *p* < 0.001, P14 *p* < 0.01, P22 *p* < 0.001), but no confirmation of potential Brn3a-mediated regulation (Fig. 9e, Additional file 1: Table S1). Selective expression of Pnkd transcripts will be described below (Fig. 14). Rims1 (regulating synaptic membrane exocytosis 1) transcript and gene expression is enriched in RGCs vs. retina, but only transcript-specific RNASeq analysis reveals Brn3a-dependent differential expression (Fig. 9f^I^-f^II^) . ISH confirms strong and significant GCL enrichment throughout postnatal development (WT retinas *p* < 0.001, Additional file 1: Table S1) and reveals some high-expressing cells in both the INL and GCL (Figs. 8f, 9f). Expression of Rims1 is high from P0-P14, and slightly decreases in the adult. Its dependency on Brn3a was apparent at P14 (*p* < 0.05) and becomes more significant in the adult (*p* < 0.001) (Fig. 9f, Additional file 1: Table S1 D-E).

### Secreted proteins (Figures 10 and 11)

RNASeq results predicted Brn3a^AP/WT^ RGCs enrichment and Brn3a regulation for Fam19a4 (family with sequence similarity 19, member A4) (Fig. 11d^I^-d^II^), however ISH showed only low level of GCL staining exclusively at P3-P7 without visible regulation by Brn3a (Fig. 10a). The only detectable Nptx1 (neuronal pentraxin 1) RefSeq transcript (Fig. 11a^II^) is expressed in all retina samples however it is significantly enriched in Brn3a^AP/WT^ RGCs and regulated by Brn3a, which is in agreement with RNASeq gene level analysis (Fig. 11a^I^). ISH largely confirms these findings, showing intense GCL staining beginning with P0 and persisting into the adult (Figs. 10b, 11a). Starting at P14 intense signal also appears in the INL, in a position consistent with Amacrine cell expression (Fig. 10b), but the significant GCL enrichment persists (P14 and P22 WT retinas *p* < 0.001, Fig. 11a). The GCL signal appears to be Brn3a dependent at the later ages (P14 and P22, *p* < 0.05), as it is significantly reduced in Brn3a KO retinas (Figs. 10b and 11a, Additional file 1: Table S1 D-E). Nptx2 (neuronal pentraxin 2) is GCL enriched and Brn3a-regulated according to transcript and gene level RNASeq (Fig. 11b^I^-b^II^). In ISH (Fig. 10c) Nptx2 labels fewer cell bodies in the GCL compared to Nptx1, however it is also GCL enriched in the Brn3a WT at P3 (*p* < 0.001), P7 (*p* < 0.01) and adult (*p* < 0.01) but not regulated significantly by Brn3a (Figs. 10c and 11b, Additional file 1: Table S1). Sez6l2 (seizure related 6 homolog-like 2) has 3 transcripts with very diverse profiles of expression at P3 (NM_001252567, NM_144926 and NM_001252566, Fig. 11c^II^-c^IV^). NM_001252567 is highly expressed in both RGCs and retina, except Brn3b KO retina, and it is the only Brn3a-dependent isoform (Fig. 11c^II^). NM_144926 is strongly represented in Brn3a RGCs and, surprisingly, in Brn3b KO retina, and is not regulated by Brn3a (Fig. 11c^III^). NM_001252566 expression is evenly distributed between RGCs and retina, with some enrichment in Brn3a RGCs (Fig. 11c^IV^). Gene level analysis does reveal Brn3a RGC enrichment, but it does not demonstrate any Brn3a dependency (Fig. 11c^I^). ISH using a 3’-UTR probe common for all three isoforms shows GCL enrichment throughout the larger part of postnatal development (P0-P14, Figs. 10d, 11c, Additional file 1: Table S1) and significant signs of Brn3a regulation in the GCL at P0 and P7 (*p* < 0.05). However, in the adult the GCL signal in Brn3a WT and KO retinas is indistinguishable (Figs. 10d, 11c).

### Adhesion molecules and other transmembrane proteins (Figures 12 and 13)

Both transcript and gene level RNASeq analyses demonstrated that Cdh4 (cadherin 4) is significantly enriched in Brn3a^AP/WT^ RGCs in comparison to all other retinal and RGC samples, including Brn3a^AP/KO^ RGCs, suggesting regulation by Brn3a in specific RGC populations (Fig. 13a^I^-a^II^). ISH reveals that Cdh4 is already expressed and GCL enriched at P0 (Figs. 12a, 13a), the expression increases at P3, and it is sparse in both GCL as well as INL. The distribution of INL staining is consistent with horizontal cell and amacrine cell expression (Fig. 12a). The number of Cdh4^+^ cells in the Brn3a KO GCL is consistently smaller compared to Brn3a WT throughout postnatal development, and the quantitation confirms it for the majority of studied ages (P0 *p* < 0.001, P3 *p* < 0.05, P14 *p* < 0.01, P22 *p* < 0.05, Additional file 1: Table S1). According to our RNASeq results Pcdh20 (protocadherin 20) is enriched in Brn3a^AP/WT^ RGCs relative to other RGC and retina samples, although its general level of expression is comparatively low (Fig. 13b^I^-b^II^). It is decreased in both Brn3a^AP/KO^ and Brn3b^AP/KO^ RGCs, suggesting regulation by both transcription factors, although, due to its low expression level (< 2 FPKM for the transcript) in Brn3b^AP/WT^ RGCs Pcdh20 was not selected as a Brn3b regulated gene in the screen. ISH showed very low levels of expression from P0 to P7, and a modest GCL enrichment (KO *p* < 0.001, WT not significant) and differential regulation by Brn3a (*p* < 0.05) at P14 (Figs. 12b, 13b, Additional file 1: Table S1 D). Both gene and transcript RNASeq analyses show that Rtn4rl2 (reticulon 4 receptor-like 2) is rather homogeneously distributed between RGCs and the retina (Fig. 13c^I^-1c^II^). Transcript analysis also reveals a high differential between Brn3a WT and KO RGCs (Fig. 13c^II^). ISH shows that Rtn4rl2 is GCL enriched early (P0-P3), and then the distribution changes after P7 and the level in the INL becomes significantly higher than in the GCL (P7 WT *p* < 0.001, KO *p* < 0.01, Figs. 12c, 13c, Additional file 1: Table S1 C). Tmem25 (transmembrane protein 25) has only one RefSeq transcript expressed in the retina. Despite both transcript and gene level RNASeq analyses show enrichment in Brn3a WT RGCs, the gene level diagram does not show regulation by Brn3a in RGCs probably due to the contribution of non-RefSeq transcripts (Fig. 13d^I^-d^II^). In situ developmental profile shows GCL enrichment of the expression at P0 (WT *p* < 0.05), P14 (WT *p* < 0.001) and P22 (WT *p* < 0.001), but no considerable signs of Brn3a-mediated regulation (Figs. 12d, 13d, Additional file 1: Table S1). Tmem91 (transmembrane protein 91) has two transcript isoforms (NM_001290497 and NM_177102, Fig. 13e^II^-e^III^), both of them show a modest expression in retina and RGCs. NM_001290497 is differentially expressed in Brn3a^AP/WT^ RGCs compared to Brn3a^AP/KO^ RGCs, NM_177102 is not, and probably because of this gene level analysis does not reveal differential regulation by Brn3a (Fig. 13e^I^). ISH confirms expression of Tmem91 starting at P0-P3, increasing through P7-P14, and diminishing in P22 (Fig. 12e). It also shows a significant differential between Brn3a WT and Brn3a KO GCL expression at P7 (*p* < 0.001) and P14 (*p* < 0.01) (Figs. 12e, 13e, Additional file 1: Table S1 C-D).

### Combined RNASeq, RT-PCR and ISH analysis for specific transcript isoform expression

Many of the described candidate genes (Figs. 2, 3, 4, 5, 6, 7, 8, 9, 10, 11, 12 and 13), selected based on the presence of at least one transcript enriched in Brn3a^AP^ RGCs and regulated by Brn3a, had several transcript isoforms expressed in the retina, often showing distinct expression patterns. The ISH tests performed with 3’-UTR probes common to all transcripts (Table 1) did therefore report a combination of the expression patterns and levels of these different transcripts, thus reflecting essentially a “gene level” expression profile. Whereas it is important to understand the expression pattern of our candidates at gene level, some biologically relevant functions could be dependent on the expression of specific isoforms. We therefore decided to further investigate the differential expression for transcripts of two genes predicted to have multiple retina-specific isoforms, Pnkd and Clcc1.

### Pnkd transcripts detected in the retina

According to RNASeq data, 3 Pnkd transcripts are expressed broadly among retina and Brn3^AP^ RGC samples (Fig. 14a-b). The long isoform (NM_001039509), initiated at exon 2, and skipping exons 4 and 5 (green splicing sequence, Fig. 14b) is enriched in both Brn3a^AP/WT^ and Brn3b^AP/WT^ RGCs, but only regulated by Brn3b. The “medium” transcript (NM_019999), predicted to start at exon 5 (purple splicing sequence, Fig. 14b), is enriched in RGCs but regulated by Brn3a. The “short” transcript isoform (NM_025580), spans three exons (e2, e3 and e4, blue splicing sequence, Fig. 14b), and shows no enrichment in RGCs. A further putative transcript, initiating at exon 1, was not detected by RNASeq in the retina or RGCs (gray splicing sequence, Fig. 14b). We sought to confirm the existence of these alternative transcripts by using RT-PCR analysis from P3 retina RNA (Fig. 14b, c; Tables 1 and 2). Bands for all positive reactions were cloned and confirmed by sequencing.

The reaction of primers 1 and 2, common to the short (NM_025580) and long (NM_001039509) transcripts, resulted in the expected product length, equal to combined exons 2 and 3. However, primers 3 + 4, recognizing exon 4, specific for the short isoform, did not produce any RT-PCR fragment, suggesting that this transcript (NM_025580) is not present in P3 retina. Primer combinations 5 + 6, and 5 + 7, specific for the exon 5 or the sum of exons 5 and 6 respectively, are selective for the medium isoform (NM_019999). Both reactions resulted in the correct product sizes (Tables 1 and 2, Fig. 14b, c). In summary, the RT-PCR analysis suggests that the long (NM_001039509) and medium (NM_019999) transcripts are expressed at detectable levels in the retina, while the short (NM_025580) transcript is not. Finally, to look at the temporal and spatial distribution of Pnkd isoforms in the retina, we generated isoform specific probes covering the long (Pr1 + Pr2), medium (Pr5 + Pr6) and short (Pr3 + Pr4) transcripts and screened our panel of postnatal retina sections (Fig. 14b, d). The long isoform is expressed at low levels in a GCL-specific manner beginning with P3 and continuing into adult (Fig. 14d, left). The medium transcript is highly expressed in the GCL from P0 to P22, but INL expression picks up at P3 and persists into the adult (Fig. 14d, middle). There is little or no Brn3a regulation for either the long or medium isoform. The short isoform was not observed in either Brn3a WT or KO retinas from P0 to P22, right column. It should be noted that the signal intensity for the medium isoform is much stronger than the one seen for the 3’-UTR probe (Fig. 8e), that should be common for both long and medium (but not short) isoforms, but the differential between signal intensity in GCL and other retinal layers is conserved between these two probes. Taken together these results suggest that Pnkd is expressed in the retina, predominantly in the GCL, but also in the INL. Whereas the enrichment of long and medium isoforms in the GCL, predicted by RNASeq data, is confirmed, the relative level of expression of the transcripts, as judged by ISH, is not entirely consistent with the RNASeq predictions. Moreover, the ubiquitous retinal expression of the short isoform, predicted by RNASeq, is not confirmed by either RT-PCR or ISH.

### Clcc1 transcripts detected in the retina

**Fig. 15:**
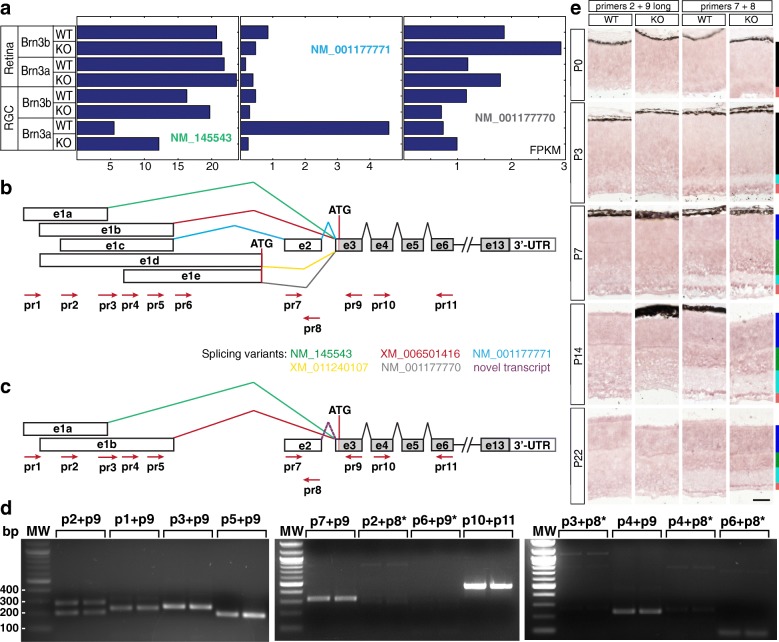
Clcc1 transcripts detected in the retina. **a** RNAseq profiles expressed in FPKM of the three Clcc1 transcripts detected in retinal and RGC-derived samples of Brn3a and Brn3b WT and KO mice at P3. **b** Predicted Clcc1 splicing variants. Exon and splicing connection annotations (labelled as in Fig. 14), for five distinct transcript variants are annotated: NM_145543, NM_001177771, NM_001177770, XM_006501416, XM_011240107. Red arrows show the positions of diagnostic primers (see also Table 2). Translation start sites are in exons e1d, e1e and e3 (ATGs indicated). **c** Confirmed Clcc1 splicing variants. Purple line indicates a novel transcript suggested by our RT-PCR analysis. **d** RT-PCR reactions from P3 retina RNA. Primer pairs as in **b**. Pairs marked with a star indicate negative or nonspecific (weak bands of incorrect size) reactions. Duplicates are shown for each primer combination. For expected product sizes, see Table 2. All bands generated by RT-PCR were gel-extracted, subcloned and sequenced for confirmation. **e** in situ hybridization in retinal sections from WT and Brn3a-KO retinas at five developmental stages. RNA probes were targeted against exons that are specific for different transcript variants. For pr2 + pr9, the probe was generated from the longer product (see panel **d**), and is predicted to detect both NM_145543 (green) and XM_006501416 (red) isoforms. Probe generated from pr7 + pr8 targets exon2 and therefore detects the newly identified transcript. Scale bar – 50 μm

GeneBank annotation predicts that Clcc1 has five transcript isoforms, consisting mostly of alternative, partially overlapping sequences for exon 1, resulting from 4 alternative transcription start sites and three different exon1 3′ ends: NM_145543 – exon 1a, XM_006501416 – exon 1b, NM_001177771 – exon 1c, XM_011240107 – exon 1d, NM_001177770 – exon 1e. Exon 2 is included only in transcript NM_001177771 (Fig. 15b). Our RNASeq data predicted three retinally expressed Clcc1 transcripts: NM_145543, NM_001177771 and NM_001177770 (Fig. 15a, b). NM_145543 (Fig. 15b, green splicing sequence) is highly expressed in RGCs and retina, with lowest expression levels in Brn3a^AP/WT^ and Brn3a^AP/KO^ RGCs (Fig. 15a, left). In contrast, NM_001177771 (Fig. 15b, blue splicing sequence) is considerably expressed only in Brn3a^AP/WT^ RGCs (Fig. 15a, middle), predicting both specificity for Brn3a RGCs and regulation by Brn3a. Finally, NM_001177770 (Fig. 15b, gray splicing sequence) is expressed moderately in both retina and RGCs (Fig. 15a, right). Given the large degree of overlap between Clcc1 isoforms, and the presence of potential alternative open reading frames, we were interested to map out the precise boundaries of exon 1 variants. We performed RT-PCR from P3 retina samples using primers targeted to specific exon regions of each transcript (Tables 1 and 2, Fig. 15b-d). All positive bands were subcloned and confirmed by sequencing. Primers 10 and 11 were used to diagnose a product spanning exons 4, 5 and 6, common to all transcript variants (~ 400 bp, Fig. 15b-d).

Forward primers Pr1 to Pr6, tilling all possible exon 1 variants, were individually paired with reverse primer Pr9, placed in exon 3, the first common exon for all splicing isoforms. Combinations of Pr1 to Pr5 with Pr9 resulted in product lengths consistent with the presence of exon variants 1a, and 1b, and excluding variants 1c, 1d and 1e (Fig. 15b-d, and see Table 2 for product lengths). E.g., the primer combination Pr2 + Pr9 gave 2 bands corresponding to XM_006501416 (exon e1b, longer fragment, ~ 300 bp) and NM_145543 (exon 1a, shorter fragment, ~ 200 bp), while combination Pr1 + Pr9 gave only one band ~ 200 bp for NM_145543 as expected. The combination of Pr6 + Pr9 showed only nonspecific bands (small amount and incorrect size), thus excluding isoforms XM_011240107 and NM_001177770 (exons 1d or 1e spliced to exon 3). All primer combinations detecting splicing sequences including exons 1 and 2 (forward Pr2, Pr3, Pr4, Pr6 combined with reverse Pr8) failed to give a positive reaction. However, splicing of exon 2 onto exon 3 is occurring, since the combination of forward primer Pr7 (5′ end of exon 2) and reverse Pr9 gave a positive reaction of expected length and sequence. Pr7 + Pr8 produced the expected size product (see below). In summary, our RT-PCR analysis narrows the number of Clcc1 transcripts expressed in the retina to NM_145543 and XM_006501416, and proposes a new alternative transcript initiating at exon 2. This novel transcript would explain the reads assigned to NM_00117771, that follows the splicing sequence e1 – e2 – e3, which we could not detect in the retina. We next determined the cellular distribution and developmental profile of the retinally expressed Clcc1 isoforms by ISH. The combined expression pattern of NM_145543 and XM_006501416 was determined using a probe that spans the exon 1b to exon 3 splicing event (Pr2 + Pr9, long product), while the novel transcript variant was tested using a probe against its unique exon 2 (Pr7 + Pr8). We find that the exon 2-containing transcript is expressed in the GCL starting with P3 and the INL beginning with P7. The GCL signal persists into the adult age and shows only a modest differential in Brn3a KO vs. WT retinas (Fig. 15e, right column), while ISH signal generated by the combined expression level of NM_145543 and XM_006501416 was relatively low throughout postnatal development (Fig. 15e, left column). In summary, only three Clcc1 transcript isoforms are present in retina: NM_145543, XM_006501416, and a novel uncharacterized transcript containing exons 2 and 3. The three isoforms are predicted to encode the same protein, with the translation initiation site located in exon 3, however, they have three distinct transcription start sites.

### Immunohistochemical analysis of two potential Brn3a targets – Elfn1 and Cdh4

We have focused so far on validating expression of potential Brn3a target molecules at the mRNA level. However, we are ultimately interested in the potential roles played by the encoded proteins in RGC cell type formation. We therefore sought to determine the cell type distribution and cellular localization of some of our targets. We show here results for two adhesion molecules playing potential roles in dendrite lamination and synaptic development, Cdh4 and Elfn1, during one developmental time point (P7) and in the adult (P22).

**Fig. 16:**
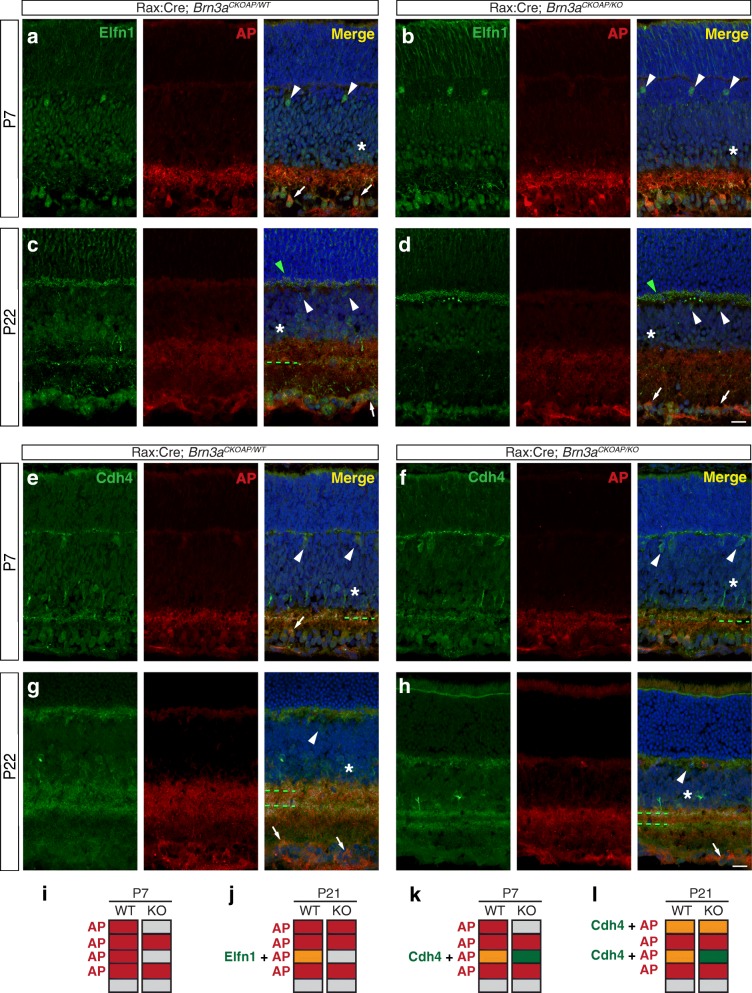
Distinct Brn3a effects on the expression of two adhesion molecules – Elfn1 and Cdh4. Retinal sections were prepared from either Rax:Cre; *Brn3a*^*CKOAP/WT*^ (**a, c, e, g**) or Rax:Cre; *Brn3a*^*CKOAP/KO*^ (**b, d, f, h**) mice at P7 (**a**, **b**, **e**, **f**) or P22 (**c**, **d**, **g**, **h**). Immunostaining for either Elfn1 (green, **a-d**) or Cdh4 (green, **e-h**) was performed in conjunction with immunostaining for Brn3a^AP^ RGCs (red, AP) and nuclear layers counterstain (DAPI, in blue, in merge channel). For every immunostaining Elfn1 (left), AP (middle) and merge (right) channels are shown. White arrowheads, asterisks and arrows indicate Horizontal Cells, Amacrine Cells and RGCs. Green arrowheads in C and D reveal synaptic staining in the OPL, as previously reported [81]. Green stippled lines in the merge channel indicate lamination of Elfn1^+^ or Cdh4^+^ neurites in the IPL, if present. Scale bar in H = 50 μm. **i-l** Schematics of Brn3a^AP^ RGCs dendrites (red) and Elfn1 (orange, I, J) and Cdh4 (green or orange, K, L) lamination in the IPL, at P7 (I, K) or P22 (J, L), in Rax:Cre; *Brn3a*^*CKOAP/WT*^ (left, WT) or Rax:Cre; *Brn3a*^*CKOAP/KO*^ (right, KO) retinas. Elfn1 stains only a few diffuse dendrites at P7, however a sharp lamina is labelled in P22, and this lamina is absent from Brn3a null retinas. Cdh4^+^ dendrites form one clear lamina at P7 and two distinct laminae at P22, however the lamination pattern is not affected by Brn3a ablation

At P7, Elfn1 expression is found in RGCs, Amacrine, Horizontal cells and Photoreceptors (Fig. 16a-b). Some Elfn1^+^ RGCs (identified by co-labelling with AP generated from the recombined *Brn3a*^*AP*^ allele) show intense staining in proximal dendrites. By P22, intense and punctate Elfn1^+^ staining is visible in the OPL (Fig. 16c-d). A distinct Elfn1^+^ lamina is visible in the IPL of Brn3a WT retinas, but essentially absent in Brn3a KO retinas. This suggests that at least one Elfn1^+^ cell type, presumably an RGC, is either deleted or has an altered morphology in Brn3a KO retinas. Alternatively, Elfn1 could be under Brn3a transcriptional control (Fig. 16c, d, i, j).

Cdh4 is expressed at P7 in RGCs, Amacrine and Horizontal cells. Strongly labeled Cdh4^+^ cell bodies in the INL most likely belong to Amacrine and Horizontal cells and are observed at both P7 and P22 (Fig. 16e-h). RGC staining does not seem to persist in the adult Brn3a WT retina (Fig. 16g) One Cdh4^+^ lamina is present in the IPL at P7 (Fig. 16e-f), while two laminae appear intensely Cdh4^+^ in the adult (Fig. 16g-h). None of these laminae is significantly affected by Brn3a ablation at either P7 or P22 (Fig. 16g, h, k, l). Unlike for Elfn1, a strong OPL Cdh4^+^ positive staining emerges already at P7. In conclusion, whereas both Elfn1 and Cdh4 were somewhat enriched in RGC during early postnatal development, adult patterns of expression include multiple other cell types, and RGC expression of Elfn1 appears profoundly regulated by Brn3a in the adult stage, while Cdh4 RGC expression levels decline to undetectable levels in both WT and Brn3aKO GCL.

## Discussion

We report here the results of a screen for genes potentially involved in mediating the effects of transcription factor Brn3a in RGC type specification. The screen covered 5 postnatal ages, between P0 and adult, and employed ISH, RT-PCR, and immunohistochemistry. The genes were selected using data from a previously published RNASeq screen for Brn3a candidate target genes in P3 RGCs [5], and had to have at least one transcript satisfying the following three criteria: 1) expression of at least 2 FPKM in Brn3a^AP^ RGCs; 2) expression enrichment of two-fold in Brn3a^AP/WT^ RGCs compared to the retina; 3) downregulation of at least two-fold between Brn3a^AP/WT^ and Brn3a^AP/KO^ RGCs. From the 180 transcripts satisfying these criteria in the original screen, we narrowed our search to genes whose molecular structure suggested potential functions in cell type specification, i.e. transcription, translation, intracellular signaling, synapse formation and function, vesicular transport or release, secreted and cell surface molecules. Of the 28 selected genes, a majority (20/28) showed increased GCL signal compared to the retina, but only a small fraction (4/28) exhibited significant Brn3a regulation at P3 (Table 3). Moreover, the expression profile of most candidate genes was extremely dynamic during postnatal development, and only a handful exhibited RGC enrichment and/or Brn3a regulation throughout all developmental ages.

**Table 3:** The expression of Brn3a target genes: ISH and RNASeq results comparison

Gene	Known function	GCL P3	GCL P0-P22	Brn3a target P3	Brn3a target any age	Postnatal GCL Expression			GCL Sparse	Brn3a-independ. Transcripts	Brn3a-regulation Gene level
						Onset	Peak	Offset			
Transcriptional and translational regulators											
Foxp2	TF	yes	no	no	yes	P0	P7	no	no	no	yes
Tshz2	TF	yes	yes	yes	yes	P3	P7	no	no	no	yes
Rbfox1	Splicing of neuronal genes	yes	no	no	no	P0	P14	no	no	yes	no
Intracellular signaling and cytoskeleton-associated genes											
Eml1	Microtubule-binding protein	yes	no	no	no	P0	P7	no	no	yes	no
Hpca	Calcium sensor	yes	yes	no	yes	P0	P7-P14	no	no	yes	no
Plppr3	Neurite growth and regeneration	yes	yes	no	yes	P0	P0	no	no	no	no
Mapk10	Axonal growth and neuronal survival	yes	yes	no	yes	P0	P7-P14	no	yes	yes	no
Pip5kl1	Cell morphology and adhesion	no	no	no	no	P3	P7	no	no	no	no
Pak6	Signaling kinase	no	no	no	no	no	no	no	no	no	na
Vesicle-associated proteins											
Ankrd13b	Caveolin- mediated endosomal traffick	yes	yes	no	no	P0	P7	no	no	no	no
Pick1	Glutamate receptor trafficking	yes	no	no	no	P0	no	no	no	yes	no
Snap91	Clathrin-mediated endocytosis	yes	yes	yes	yes	P0	P7-P14	no	yes	yes	no
Tusc5	Insulin-stimulated glucose transport	yes	yes	yes	yes	P3	P14	no	yes	no	yes
Synapse-associated proteins											
Elfn1	Synaptic adhesion protein	yes	no	no	yes	P3	P7	no	yes	no	yes
Gabra1	GABA receptor alpha1 subunit	no	no	no	yes	P3	no	no	yes	no	yes
Grm4	Metabotropic glutamate receptor	yes	no	no	no	P0	P7	no	no	no	yes
Ntrk1	Neurotrophin receptor	yes	no	no	no	P0	P14-P22	no	no	no	yes
Pnkd	Synaptic vesicle release	no	yes	no	no	P0	P7-P14	no	yes	yes	no
Rims1	Synaptic vesicle release & recycling	yes	yes	no	yes	P0	P0-P3	no	yes	no	no
Secreted proteins											
Fam19a4	Unknown	no	no	no	no	P3	P3-P7	P7	no	no	yes
Nptx1	Trans-synaptic, excitatory synapse	yes	yes	no	yes	P0	P14	no	yes	no	yes
Nptx2	Trans-synaptic, excitatory synapse	yes	no	no	no	P3	P7-P14	no	yes	no	yes
Sez6l2	Synapse formation	yes	yes	no	yes	P3	P7	no	no	yes	no
Adhesion molecules and other transmembrane proteins											
Cdh4	Cell adhesion molecule	yes	no	yes	yes	P0	no	no	yes	no	yes
Pcdh20	Cell adhesion molecule	no	no	no	yes	P14	no	no	no	no	yes
Rtn4rl2	Axon guidance molecule	yes	yes	no	yes	P0	no	P22	no	no	no
Tmem25	Unknown	no	no	no	no	P0	P7-P14	no	no	no	no
Tmem91	Unknown	no	no	no	yes	P0	P7-P14	no	no	yes	no

Column 1 represents gene name, column 2 – known function of the gene, column 3 – GCL enrichment at P3, column 4 – consistent GCL enrichment (at least at 4 out of 5 ages) from P0-P22. Fifth column shows whether the gene is regulated by Brn3a at P3, 6th – regulated by Brn3a at any of 5 studied ages. Columns 7, 8 and 9 represent the onset, the peak and the offset respectively of postnatal gene expression in the GCL. Column 10 shows whether the gene is highly and sparsely expressed in the GCL at any of studied ages. Columns 11 and 12 identify the genes that had unregulated transcripts or exhibited gene level regulation by RNASeq

### Differential expression: RNASeq, ISH and developmental dynamics

What molecules with potential to influence RGC type specificity are Brn3a targets? The gene expression profiling screen at the origin of this study was focused on postnatal day 3 and looked at Brn3a RGC specific transcripts. Our reasoning was that P3 is a particularly active time point for neurite branching and synapse formation for both RGC dendrites and axons. However, RGCs, which become postmitotic beginning with E12.5, are probably amongst the most mature neurons in the early postnatal retina. Thus, by selecting genes that had transcripts with higher expression levels in Brn3a RGCs versus retina supernatants, it is likely that we included molecules enriched in mature neurons. In addition, transcript and gene level RNAseq analysis reveals that many of our target genes had RGC specific as well as more broadly expressed transcripts. Thus, using probes placed in the 3’UTR, typically recognizing most transcripts of a gene, will necessarily miss some of the differences predicted by RNASeq at the transcript level. Nevertheless, it is important to know the overall gene expression levels for any given gene in the retina and RGCs, in order to better predict and understand phenotypic changes upon loss of function manipulations. Taken all these considerations into account, it is rewarding to find that gene-level ISH confirmed RGC enrichment for about two thirds of the targets (20 of 28, 71% accuracy), and slightly more than half for Brn3a dependent RGC expression at any given age (16 of 28, 57% accuracy). A subset of our targets, (Rbfox1, Eml1, Hpca, Mapk10, Pick1, Pnkd, Sez6l2, Tmem91) had multiple RefSeq transcripts, some of which were not Brn3a dependent; with gene level RNASeq analysis did not show Brn3a dependency. This could explain why, for all these genes, ISH using the 3’UTR did not show differences of expression at P3. It is however worth mentioning that Mapk10 appeared Brn3a dependent at all other studied ages. Another 16 targets (Foxp2, Plppr3, Pip5kl1, Pak6, Ankrd13b, Elfn1, Gabra1, Grm4, Ntrk1, Rims1, Fam19a4, Nptx1, Nptx2, Pcdh20, Rtn4rl2, Tmem25) have only one expressed transcript each, predicted to be Brn3a-dependent. Exactly half of those genes (8 of 16) seem to be significantly regulated at postnatal ages other than P3 (Foxp2 – P7, P22; Plppr3 – P14; Elfn1 – P14-P22; Gabra1 – P22; Rims1 – P14-P22; Nptx1 – P14-P22; Pcdh20 – P14; Rtn4rl2 – P7), but another half (Pip5kl1, Pak6, Ankrd13b, Grm4, Ntrk1, Fam19a4, Nptx2, Tmem25) do not reveal any signs of Brn3a-mediated regulation at any stage of postnatal development. Of note, the presence of Brn3a independent transcripts was a strong predictor of lack of Brn3a regulation at gene level. However the presence of only one, Brn3a dependent RefSeq transcript was not necessarily linked with gene level regulation, or confirmation by ISH, most likely suggesting the existence of further, non-RefSeq transcripts (e.g. Pip5kl1, Ankrd13b and Tmem25).

Among the molecules that were confirmed by ISH to be Brn3a-dependent at P3 we found mRNAs for transcription factor Tshz2, vesicle-associated proteins Snap91 and Tusc5, and adhesion protein Cdh4. While Tshz2 and Snap91 mRNAs show only transient Brn3a dependency, Tusc5 and Cdh4 represent developmentally consistent Brn3a regulation targets (Table 3). Mapk10 showed fairly consistent Brn3a-dependency (with the exception of P3, Figs. 4d, 5d). Brn3a regulation could be hidden by Brn3a-negative RGC types, or by non-RGC expression in the ganglion cell layer which derives from Amacrine cells. In case of genes regulated by Brn3a early (P3-P7), but not late (P14-P22) (for instance, Snap91), delayed expression could occur in Brn3a-independent RGC subtypes or in Amacrine cells. A subset of targets (Elfn1, Gabra1, Rims1, Nptx1 and Pcdh20) which are GCL-expressed and Brn3a-regulated at later ages (P14-P22), are potentially more important during RGC synapse maturation in the retina after eye-opening and for Brn3a-expressing RGC function. Our findings suggest that patterns revealed by RNA sequencing may only partially be reproduced by other detection techniques. In addition, many genes may change their pattern of expression over time, especially when samples are derived from rapidly developing tissues, such as early postnatal retina. These dynamic expression profiles could also suggest the developmental steps at which the various target genes might be involved, as described below.

### Transcript level analysis of cell type specificity

Many of the candidate genes identified in our RNAseq screen had two or more transcripts, with mixed patterns of RGC enrichment and/or Brn3a regulation. We therefore tested the accuracy of transcript level analysis and its influence on diagnosing cell type specificity of gene expression for two candidate genes – Pnkd and Clcc1, using transcript-specific RT-PCR reactions and ISH probes. We chose Pnkd since it has three previously characterized transcripts, encoding different protein isoforms, with the long version, Pnkd-L (long transcript) thought to be CNS-specific [52]. For Pnkd (3 predicted retinal transcripts - long, medium and short), only one transcript was predicted by RNASeq to be Brn3a-dependent. Both RT-PCR and ISH confirmed the RGC enrichment of the Pnkd medium transcript suggested by RNAseq for P3 retina. However, there was a strong discrepancy between the relative abundance of the long and medium size transcripts as reported by RNASeq and ISH, and the third, short isoform was undetectable by either RT-PCR or ISH. Furthermore, the ISH signal intensity was far stronger for the medium transcript specific probe compared to the 3’UTR probe detecting all isoforms, although hybridization strength based on base pair composition and probe length (360 bases for 3’-UTR and 167 bases for medium transcript) favor the 3’UTR probe. Clcc1 has an unusually large number of exon 1 variants – based on different transcription start sites and exon 1 splice donor sites. Also, Clcc1 transcript NM_001177771 was predicted to be highly expressed specifically in Brn3a WT and not in Brn3a KO RGCs or other parts of retina. Clcc1 has 5 alternative transcripts, mostly based on alternative transcription start sites at exon 1. Of the three predicted retina specific variants, one (NM_001177771) was supposed to be highly differentially expressed in Brn3a^AP/WT^ RGCs and regulated by Brn3a. Our RT-PCR analysis with transcript-specific primers did not confirm the expression of this isoform, but rather suggests the presence of a novel, closely related transcript initiated at exon 2, and splicing onto exon 3. This novel isoform was found to be GCL-enriched by ISH at P3 but expanded its expression to the INL from P7 to P22. The encoded protein should be the same for NM_001177771 and the novel isoform since the open reading frame starts at exon 3, shared by both. Moreover, long exon 1 variants (NM_001177770 and XM_011240107) were not confirmed in our experiments, suggesting that there is no basis for existence of alternative protein variants with an open reading frame start at exon 1. Taken together, the outcomes of the transcript level analyses for Pnkd and Clcc1 raise several points to be considered when evaluating RNASeq predictions of cell type specificity. Transcript level analysis by RNASeq is constrained to aligning reads onto the existing transcript annotations, and hence relies on their quality. In addition, it could misrepresent the relative abundance of existing transcripts if it relies on reduced read numbers that do not span exon boundaries. In the case of Clcc1, the challenge was particularly steep given the large number of annotated alternative transcription start sites and splice donor sites at exon 1. Since most RNASeq data are derived from mRNA by using poly-dT capture of the polyA at the 3′ end, this last issue could be particularly severe at the 5′ end of the mRNA, as seen for both Pnkd and Clcc1. RNASeq results could be biased by amplification artefacts, while ISH detection could be influenced by the specific sequence properties of the used probes, as seen for the Pnkd medium isoform. Although, for both tested genes, a careful transcript level analysis did explain the RNASeq predictions, our data argues for using the deep sequencing predictions as a starting point for more careful transcript characterization.

### Transcription and translation – related targets

The two identified transcription factors, Foxp2 and Tshz2 [53, 54] are expressed and GCL-enriched early in postnatal development (P0-P7), however at P14 – P22 Tshz2 expression is reduced but limited to the GCL, while Foxp2 expression extends to include both INL and GCL. This is somewhat surprising since recent reports suggest that retinal Foxp2 expression is restricted to a subset of RGC types [55, 56]. However, a transiently broader pattern of expression is suggested by using a Foxp2^Cre^ crossed into a general reporter [55]. Although Foxp2 missense mutations are associated with speech impairment in humans, the gene has clearly a broader developmental role, as mouse loss of function alleles result in lethality at 3–4 weeks of age [57]. Tshz2 is one of the three homologues of the Drosophila transcription factor teashirt, a gene involved in the homeotic control of body segment and imaginal disc formation that cooperates with eyeless (Pax6) in establishing fly retina identity [54]. Tshz2 has not been studied in great detail, however, mouse ISH data reveals cell type specific localization in several brain regions [58], suggesting a role in cell type specification. Thus, both identified TFs are good candidates for mediating Brn3a roles in RGC type specification. The splicing regulator Rbfox1 is enriched but not restricted to the GCL beginning with P3, and eventually expands to the whole retina by adult age. This could reflect a role in neuronal maturation, by regulating splicing of mRNAs for proteins implicated in synapse transmission and plasma membrane potential formation [59], especially during the period of active synaptogenesis (P3-P14) when its level is high according to our data.

### Intracellular signaling components

We characterized the expression of five genes involved in intracellular signaling cascades. Eml1 is a microtubule-binding protein, and its disruption results in the generation of ectopic neuronal progenitors in the cerebral cortex of both mouse and human [60]. Interestingly, the fish homologue of Eml1 is expressed in photoreceptors and mediates Ca^2+^ modulation of the cyclic GMP gated channel [61, 62]. Hpca is a calcium sensor protein modulating slow afterhyperpolarization in the brain [63], but with many other suggested functions in morphogenesis. Mapk10 encodes the signaling molecule Jnk3 implicated in axonal growth and regeneration and neuronal survival [39, 64]. Plppr3 is a member of the lipid phosphate phosphatase-related protein family, which share sequence homology with known phosphatases but are catalytically inactive due to substitutions in known key aminoacids in the catalytic domains. They are five pass transmembrane proteins, were shown to stimulate membrane protrusions and neurite growth in cell lines and primary neuron culture and facilitate axonal outgrowth and regenerative sprouting [65, 66]. Pip5kl1 is a brain-specific, kinase-dead isoform of phosphatidil-inositol-(4)-phosphate 5-kinase, operating as a scaffold for recruitment of other phosphatidil inositol 5 – kinases, and implicated in cell morphology control and adhesion foci [67]. Eml1, Hpca, and Mapk10 were all expressed in the GCL beginning with P0 with a peak around P7-P14, and persistence into the adult. Their early and persistent pattern of expression is consistent with participation in both molecular and activity dependent processes, beyond the period of eye-opening (~P14) [68, 69]. The two molecules involved in phospholipid signaling, Plppr3 and Pip5kl1, follow different trends. Plppr3 expression is strong already at P0, and decreases dramatically towards adult, while Pip5kl1 is expressed in the GCL, although transiently (P3-P7) and at low levels. This expression profile suggest roles in the early stages of dendrite formation and/or axon branching.

Three of the genes encoding vesicle-associated proteins – Ankrd13b, Pick1 and Snap91 – also showed early expression in the GCL (P0 – P3), with gradual expansion of the signal in both the GCL and the INL from P3 till adult age. Ankrd13b participates in caveolin-1 mediated endosomal trafficking [70]. Pick1 is involved in glutamate receptor trafficking [71], while Snap91 is implicated in clathrin-mediated endocytosis, and participates in axogenesis and dendritic growth in hippocampal neurons [72]. Tusc5 is the only gene from this group showing strong and sparse GCL signal that is Brn3a dependent, beginning with P3. It was shown before to regulate insulin-stimulated glucose transport in adipocytes [73], but it is also expressed in peripheral neurons [74] and olfactory receptors [75].

### Synapse associated molecules

The expression dynamic observed for intracellular signaling and vesicle associated proteins is largely followed by most synapse-associated genes explored in our screen, i.e. Gabra1, Grm4, Ntrk1 and Pnkd. Early (P0 or P3) GCL enrichment is followed by expansion of the expression domain into the INL towards the adult ages. Later the expression patterns diversify. Gabra1 becomes highly but sparsely expressed in the INL and GCL, and exhibits Brn3a regulation in the GCL, while Grm4 and Ntrk1 expression is broad in the INL and the GCL. The expression for both Grm4 (encoding the metabotropic glutamate receptor mGluR4 [76]) and Gabra1 (encoding the GABA (gamma amino butyric acid) receptor alpha 1 subunit) [77, 78] have been previously characterized in the retina at the protein level. Both receptors exhibit characteristic lamination profiles in the IPL. Our in situ data now shows that mGluR4 (Grm4) and Gabra1 are expressed in Bipolar, Amacrine and Ganglion cells, and thus could participate in Bipolar-Amacrine-RGC triad synapses at both pre-and postsynaptic sites, as suggested for the cerebellum [79]. The recently characterized synaptic molecule Elfn1 is also expressed pre- and post-synaptically in multiple neuronal cell types [80–82]. It presumably acts as a trans-synaptic adhesion molecule, and regulates presynaptic release probability of certain hippocampal neurons [82]. In the retina, Elfn1 was shown to participate in establishment of Photoreceptor-Bipolar Cell contacts via trans-synaptic interaction with metabotropic glutamate receptor mGluR6 [81]. Our ISH reveals high levels of Elfn1 expression in a subpopulation of RGCs beginning with P3 and through adult age, while IIF reveals Elfn1 positive cell bodies in the GCL and a narrowly stratified band in adult but not P7 IPL. Both the intensely labelled GCL cell bodies and the Elfn1 positive IPL sublamina are under Brn3a control, as they are absent from the Brn3a KO retina. In addition, immuno-detection of Elfn1 protein confirmed the previously described punctate (synaptic) pattern in the OPL [81], but also revealed expression in Photoreceptors, Horizontal, Amacrine and RGCs beginning at P7 and into the adult age. A similar pattern is revealed by Cao et al. [81] in supplementary material. These results are distinct from the ISH pattern, which confirms widespread retinal expression of Elfn1 only in the adult. This may be due to distinctions in sensitivity between ISH probe and antibody, or different stability of the mRNA versus protein.

The broad expression of Ntrk1 (encoding the neurotrophin receptor TrkA) in Bipolar, Amacrine and RGCs raises some interesting questions regarding the role of neurotrophic support in the inner retina. Whereas a role of TrkA as a RGC receptor for target derived neurotrophins originating in the retinorecipient areas has been proposed [83], the expression in many inner retina neurons after eye opening may suggest a broader role in establishing and maintaining synaptic communication in the IPL.

Pnkd exhibits a significant differential between GCL and INL especially in the adult, when the GCL pattern becomes sparse, while Rims1 is highly expressed in sparse populations in the adult, combined with lower expression level in the rest of the INL and GCL. Pnkd could be implicated in presynaptic vesicle release, by interacting with the protein encoded by Rims1 [52], a major component of the vesicle release machinery in multiple neuronal types [84, 85]. Thus Pnkd and/or Rims1 could potentially cooperate in modulating the synaptic release properties for distinct RGC subtypes.

Three genes from the secreted proteins group are also associated with synaptic function. Nptx1 (neuronal pentraxin 1, NP1) is a trans-synaptic factor for glutamate receptor subunit GluR4 recruitment to synapses, and it is secreted from presynaptic neurons [47]. The product of Nptx2 gene – Narp (neuronal activity regulated protein) is also synaptically released [86], and it is crucial for excitatory synapse maturation in hippocampus [87]. Both Narp and Nptx1 participate in synaptic refinement in developing visual system [88]. In our ISH screen, mRNAs of those two genes are sparsely and highly expressed in both the INL and the GCL, but Nptx1 seems more GCL-enriched and Brn3a-dependent compared to Nptx2 during postnatal development. Sez6l2 is a member of the “Brain-Specific Receptor-Like” family of proteins which contains single-pass transmembrane receptors whose extracellular domain can be cleaved [89, 90]. Knock-out mice for the three members of this family exhibit synapse formation abnormalities in the cerebellum, and autoantibodies against Sez6l2 are implicated in an autoimmune syndrome comprising ataxia and retinopathy [90, 91]. Sez6l2 seems to maintain a level of enrichment in the GCL from P3 to P14 in our ISH and the related Sez6l is expressed in subpopulations of RGCs and Amacrine cells [92].

The final group contains genes encoding cell surface molecules potentially involved in cell-cell communication and/or axon/dendrite growth. Cdh4 (R-cadherin) is a cell adhesion molecule which was previously shown to be expressed in the retina in RGCs, Amacrine and Horizontal cells, findings which were largely confirmed in our ISH and IIF data [93–95]. Although our ISH data also shows a modest regulation of Cdh4 by Brn3a at late postnatal ages, the two IPL laminae typically marked by Cdh4 protein [94] are not affected in Brn3a KO mice, suggesting that they are generated by Amacrine cells or Brn3a independent RGCs. We also find late developmental GCL enrichment and Brn3a regulation for Pcdh20, a protocadherin expressed in several other neuronal cell types [96, 97]. In contrast, Rtn4rl2, encoding the Nogo receptor 2, is GCL enriched only early (P0-P3), and then gets upregulated in the INL. Its exact function in neuronal development, including the visual system is still unclear [98–100]. Finally, not much is known about the two transmembrane proteins Tmem25 and Tmem91.

Our screen also revealed several molecules that are associated with neuronal activity. Several genes (e.g. Hpca and Rbfox1) have a peak of expression at P14, with decreasing abundance in the adult. This could represent the changes in almost mature retina after eye-opening, when the amounts of gene and protein expression required for arbor formation and growth are substituted with the amounts needed for maintenance of the structure and functionality.

According to our data, Brn3a regulates constitutively expressed partner of the immediate early gene Nptx2 – Nptx1 [47, 86, 87, 101, 102], as well as the GABA receptor subunit Gabra1 [103] around and after eye-opening (P14, P22), when synaptic maturation in retinal circuits occurs [104]. Assuming that this is not caused by specific, late RGC cell loss, it would argue for a role of Brn3a in functional maturation of RGCs. Those changes take place during the period of light-driven dendritic refinement, and they are morphologically subtle compared to events during first 2 postnatal weeks [10].

## Conclusions

Brn3a controls the expression of several GCL-enriched genes which encode synaptic, cell-surface and intracellular proteins. However, the extent and temporal dynamics of this regulation for molecules within different groups could vary, potentially depending on their molecular roles during specific stages of RGC subtype development (Table 3). The candidates identified by RNA sequencing of purified RGCs at a particular developmental time point have to be assessed using transcript-level analysis and throughout development, in order to identify potential RGC type markers. Most of the studied genes show GCL-enriched pattern of expression, and more than a half of them show Brn3a dependency during particular postnatal developmental time points. However, only Mapk10, Tusc5 and Cdh4 represent a developmentally consistent targets of Brn3a-mediated regulation in the mouse retina. According to Brn3a KO GCL pattern, while Mapk10 and Cdh4 expression are not entirely controlled by Brn3a, Tusc5^+^ cell bodies are almost completely eliminated from Brn3a KO retinas. Tusc5 thus could be a marker of one or a few Brn3a-expressing and -dependent RGC subtypes.

## Additional file

Additional file 1: **Table S1.** Semi-quantitative assessment of the expression of potential Brn3a target genes by ISH densitometry. (ZIP 47 kb)

## Abbreviations

AP: Alkaline phosphatase
BAC: Bacterial artificial chromosome
BSA: Bovine serum albumin
ChAT: Choline acetyl transferase
CPM: Counts per million reads
ES cell: Embryonic stem cell
FPKM: Fragments per million reads per gene kilobase
GCL: ganglion cell layer
IIF: Indirect immunofluorescence
INL: Inner nuclear layer
IPL: Inner plexiform layer
ISH: in situ hybridization
NBL: Neuroblast layer
NDS: Normal donkey serum
ONL: Outer nuclear layer
OPL: Outer plexyform layer
PBS: Phosphate buffered saline
PFA: paraformaldehyde
RGC: Retinal ganglion cell
RNASeq: RNA sequencing
RT: Room temperature
RT-PCR: Reverse transcription–polymerase chain reaction
TF: Transcription factor
3’-UTR: 3’-untranslated region
