# Supplementary material for: Postnatal developmental dynamics of cell type specification genes in Brn3a/Pou4f1 Retinal Ganglion Cells
Source: Neural Dev. 2018 Jun 29;13:15. doi: 10.1186/s13064-018-0110-0 (PMC6025728; doi:10.1186/s13064-018-0110-0)
Supplement: Supplementary file 1 — Table S1. Semi-quantitative assessment of the expression of potential Brn3a target genes by ISH densitometry. (ZIP 47 kb) [file 13064_2018_110_MOESM1_ESM.zip › SupplTable1.docx]

**Supplementary Table 1. Semi-quantitative assessment of the expression of potential Brn3a target genes by ISH densitometry.**

The table represents relative expression intensity for 26 genes estimated by ISH and represented as normalized to respective IPL (inner plexiform layer, all except P0) mean gray values of pixels from ROIs chosen in the NBL (neuroblast layer, P0-P3), ONL (P7-P22), INL (P7-P22) and GCL from retinas of animals with Brn3a WT and KO genotypes at 5 ages: PO – sheet **A**, P3 – **B**, P7 – **C**, P14 – **D**, P22 – **E**. In sheets **A** and **B**, column A represents gene name, column B – genotype (Brn3a WT or KO), column C – number of observations for NBL for the respective combination of gene and genotype, column D – number of observations for GCL, column E – mean value for NBL, column F – mean value for GCL, column G – median value for NBL, column H – median value for GCL, column I – Student-test (t-test) result for comparison of GCL level of expression with NBL level (1 – positive, 0 – negative), column J – p-value for t-test, column K – Kolmogorov-Smirnov (KS) test result for comparison of GCL and NBL levels of expression (1 – positive, 0 – negative), column L – p-value for KS test, columns M and N – result and p-value of t-test for comparison of WT and KO GCL levels for respective gene (shown only in WT row), columns O and P – result and p-value of KS test for comparison of WT and KO GCL levels for respective gene (shown only in WT row). In sheets **C**, **D** and **E**, column A represents gene name, column B – genotype, column C – number of observations for ONL, column D – number of observations for INL, column E – number of observations for GCL, column F – mean value for ONL, column G – mean value for INL, column H – mean value for GCL, column I – median value for ONL, column J – median value for INL, column K – median value for GCL, column L – t-test result for comparison of GCL and INL levels of expression (1 – pos., 0 – neg.), column M – p-value for t-test, column N – KS test result for comparison of GCL and INL levels of expression (1 – pos., 0 – neg.), column O – p-value for KS test, columns P and Q – result and p-value of t-test for comparison of WT and KO GCL levels for respective gene (shown only in WT row), columns R and S – result and p-value of KS test for comparison of WT and KO GCL levels for respective gene (shown only in WT row).
